# A strategy to disentangle direct and indirect effects on (de)phosphorylation by chemical modulators of the phosphatase PP1 in complex cellular contexts†

**DOI:** 10.1039/d3sc04746f

**Published:** 2024-01-12

**Authors:** Bernhard Hoermann, Eva-Maria Dürr, Christina Ludwig, Melda Ercan, Maja Köhn

**Affiliations:** a Faculty of Biology, Institute of Biology III, University of Freiburg Freiburg Germany maja.koehn@bioss.uni-freiburg.de; b Signalling Research Centres BIOSS and CIBSS, University of Freiburg Freiburg Germany; c Chair of Proteomics and Bioanalytics, Technical University of Munich (TUM) Freising Germany; d Bavarian Center for Biomolecular Mass Spectrometry (BayBioMS), Technical University of Munich (TUM) Freising Germany

## Abstract

Chemical activators and inhibitors are useful probes to identify substrates and downstream effects of enzymes; however, due to the complex signaling environment within cells, it is challenging to distinguish between direct and indirect effects. This is particularly the case for phosphorylation, where a single (de)phosphorylation event can trigger rapid changes in many other phosphorylation sites. An additional complication arises when a single catalytic entity, which acts in the form of many different holoenzymes with different substrates, is activated or inhibited, as it is unclear which holoenzymes are affected, and in turn which of their substrates are (de)phosphorylated. Direct target engaging MS-based technologies to study targets of drugs do not address these challenges. Here, we tackle this by studying the modulation of protein phosphatase-1 (PP1) activity by PP1-disrupting peptides (PDPs), as well as their selectivity toward PP1, by using a combination of mass spectrometry-based experiments. By combining cellular treatment with the PDP with *in vitro* dephosphorylation by the enzyme, we identify high confidence substrate candidates and begin to separate direct and indirect effects. Together with experiments analyzing which holoenzymes are particularly susceptible to this treatment, we obtain insights into the effect of the modulator on the complex network of protein (de)phosphorylation. This strategy holds promise for enhancing our understanding of PP1 in particular and, due to the broad applicability of the workflow and the MS-based read-out, of chemical modulators with complex mode of action in general.

## Abstract 2

This strategy identifies PP1 substrate candidates and pathways triggered by modulator treatment, offering a potentially broad applicability of this approach to other enzyme modulators.

## Graphical abstract

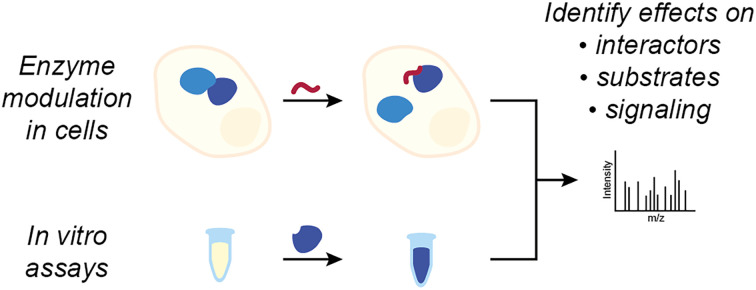

## 1 Introduction

**Fig. 1:**
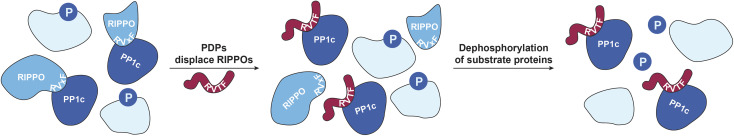
PP1 holoenzyme formation gets disrupted by PDPs. RIPPOs can bind to the RVxF-binding site on PP1c and form holoenzymes that modulate PP1c activity.^9^ PDPs (red) bind to the same RVxF-binding site and replace RIPPOs depending on their binding affinity to PP1c.^11^ The PDP–PP1c complex can dephosphorylate nearby substrates.^11,12^

Chemical modulators, in particular activators, of enzymes have become of great interest to study the respective enzymes and to target decreased enzymatic function in disease.^1–5^ Nevertheless, activators are still rather rare, and enzyme activation can have multiple downstream effects that are difficult to assess comprehensively, complicating the interpretation of enzyme activator treatments.^6^ This is particularly relevant when activating a ubiquitous enzyme such as protein phosphatase-1 (PP1), which has shown promise for alleviating cardiomyopathy phenotypes.^5,7,8^ PP1 counteracts hundreds of phospho-serine/threonine (pSer/pThr)-specific kinases selectively through the formation of holoenzymes with more than 200 known regulatory interactors of protein phosphatase one (RIPPOs) that regulate the localization and activity of the catalytic subunit of PP1 (PP1c) (Fig. 1).^9^ This complex regulation makes it difficult to assign substrates and thus, despite its ubiquity, less than a hundred substrates of PP1 have been assigned.^10^ We previously developed PP1-disrupting peptides (PDPs) that release PP1c from holoenzymes that can then dephosphorylate proximal substrates (Fig. 1).^11,12^ They were applied in end stage heart failure tissue to alleviate cardiac arrythmia,^5,7,8^ and also as bifunctional molecules, so-called phosphatase-recruiting chimeras (PhoRCs), to recruit PP1 to an oncogenic kinase in order to dephosphorylate and activate it.^9,13^ These PDPs contain a so-called RVxF-motif (with x being any amino acid) that binds to a groove on PP1c, with PDP1 containing only natural amino acids and PDP-*Nal* including the unnatural amino acid naphthylalanine and an extended basic sequence for cellular stability and uptake, respectively.^11,12^ They are selective toward PP1 over other phosphatases of the same family, and the efficacy in releasing PP1c from the holoenzyme depends on the binding affinity of the RIPPO to PP1c, thus not all holoenzymes are disrupted.^11,14^ Nevertheless, addressing the consequences of PP1 activity modulation through this mechanism comes with several challenges: How can one distinguish direct *versus* indirect dephosphorylation in intact cells since the timescale of downstream dephosphorylation events is very fast, which holoenzymes are disrupted, and does this disruption result only in dephosphorylation or possibly in higher phosphorylation levels if PP1c cannot find the substrate without a RIPPO facilitating recognition? Mass spectrometry (MS)-based technologies to study targets of drugs such as kinobeads,^15^ thermal proteome profiling (TPP),^16^ or the recent pH-dependent protein precipitation (pHDPP) approach^17^ cannot address these complex questions. This is because they identify the engaged targets, such as enzymes, of the drugs by for example their binding and stabilization, but they do not reveal the affected enzyme substrates. To address these questions, here we developed a combination of MS-based workflows to study the effects of PDP-*Nal* treatment at an early time point chosen to reduce indirect and downstream effects that accumulate over time. These approaches showed that the oncogenic protein URI1 (also known as RMP) is one of the earliest RIPPOs to be displaced by PDP-*Nal*, delivered a rich dataset of high confidence direct PDP-bound PP1 substrate candidates, and identified a new substrate.

## 2 Results and discussion

### 2.1 PP1-disrupting peptides target PP1 in a highly selective manner in intact cells

Previously, we found that neither protein phosphatase-2A (PP2A) nor calcineurin (also known as PP2B or PP3) were affected by PDPs.^11,14^ However, off-target binding to other proteins also needs to be evaluated in an untargeted, proteome-wide manner to understand PDP action in cells. To determine how selective PDPs are in a cellular context, we performed immunoprecipitation (IP) experiments in analogy to kinobeads for kinases,^15^ and analyzed proteins bound to the active peptide PDP1 as the canonical sequence^11^ and the inactive control PDP1m. The control peptide PDP1m was used to ensure that any observed changes are caused by the interaction between active PDPs and PP1c rather than other proteins. While the control peptide PDP1m lacks two amino acids within the RVxF motif required for binding to PP1c (the sequence is RATA instead of RVTF), both peptides share sequences of ten and seven amino acids at the N- and C-terminus, respectively. These sequences could in theory interact with other cellular proteins and lead to changes falsely attributed to PP1c, if only PDP1 and a vehicle control were compared.

**Fig. 2:**
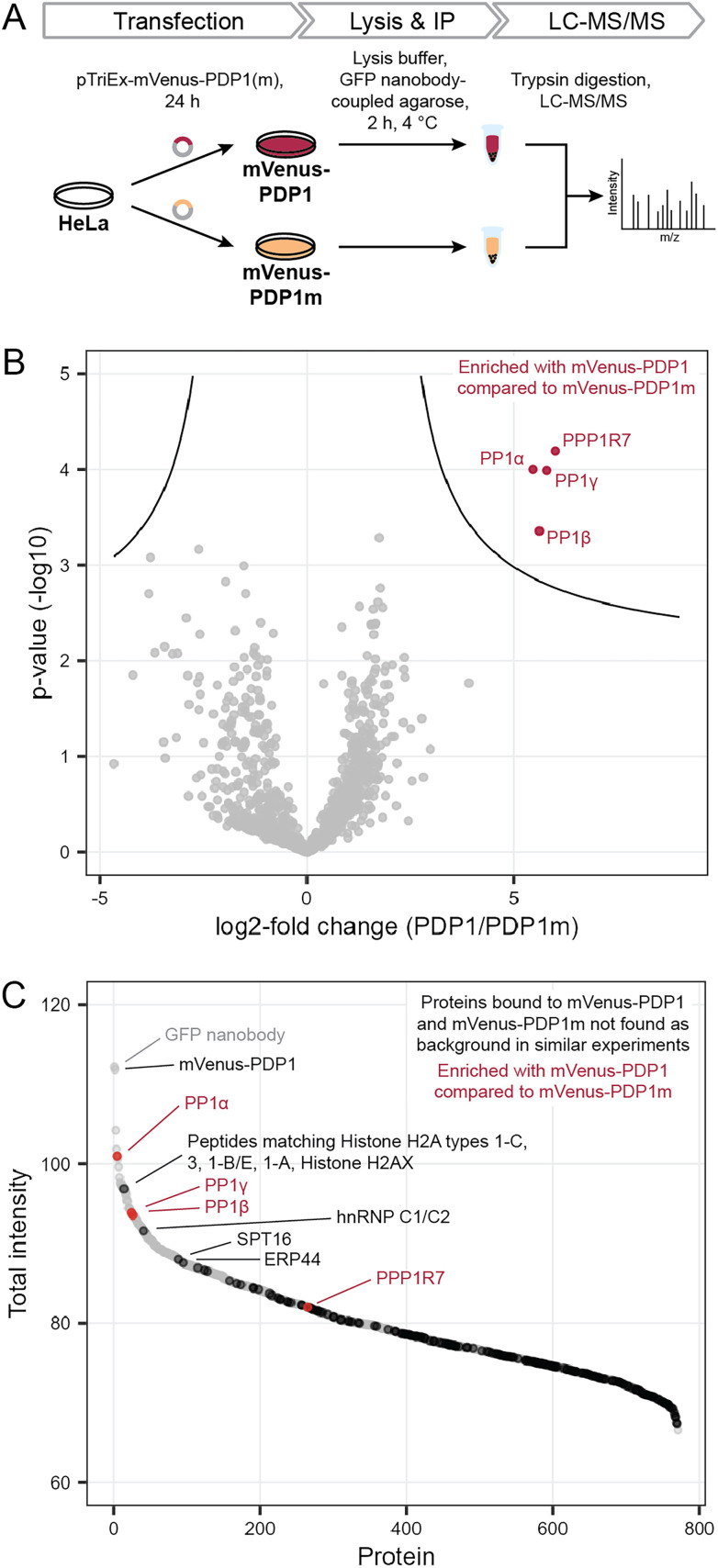
Determination of PDP specificity. (A) Workflow to assess the selectivity of PDP1 in cells using immunoprecipitation and mass spectrometry (MS). PDP1: RPKRKRKNARVTFAEAAEII, PDP1m: RPKRKRKNARVTFAEAAEII. (B) Results of MS experiment shown in A, comparing proteins bound to mVenus-PDP1 and mVenus-PDP1m. Proteins significantly enriched with mVenus-PDP1 compared to mVenus-PDP1m are highlighted in red. Significance threshold: FDR = 0.01, s0 = 0.5. (C) Sum of MS intensities of proteins bound to mVenus-PDP1. Proteins significantly different between mVenus-PDP1 and mVenus-PDP1m are highlighted in red, proteins that are not found in other affinity purification MS experiments using HeLa cells^19^ in black, with proteins among the 100 highest MS intensities labeled.

To perform the IP, we exploited the peptidic nature of PDPs and tagged the 20-amino acid peptide PDP1 (RPKRKRKNARVTFAEAAEII) and its inactive counterpart PDP1m (RPKRKRKNARATAAEAAEII) with the fluorescent protein mVenus.^18^ Sequences encoding the two peptides were cloned into a pTriEx-mVenus host vector and HeLa Kyoto cells were transfected transiently. After 24 h, cells were lysed and proteins bound to mVenus-PDP1 or mVenus-PDP1m were identified using IP of mVenus with anti-GFP beads followed by tryptic digestion and liquid chromatography with tandem mass spectrometry (LC-MS/MS) using label-free quantification (Fig. 2A).

Following normalization, filtering and imputation, a total of 1056 proteins were identified and compared between mVenus-PDP1 and mVenus-PDP1m using a Student's *t*-test (ESI Table 1†). Among the 1056 proteins only four proteins were significantly more enriched with mVenus-PDP1 compared to mVenus-PDP1m conditions (Fig. 2B). These were found to be all three isoforms of PP1c (PP1α, PP1β and PP1γ, encoded by PPP1CA, PPP1CB and PPP1CC, respectively) present in HeLa Kyoto cells and PP1 regulatory subunit 7 (PPP1R7), also known as ‘suppressor of Dis2 mutant 2’ (Sds22). While almost all known PP1 regulatory subunits bind the catalytic core protein PP1c through a combination of short linear motifs (SLiMs) situated in unstructured regions, with the vast majority utilizing the RVxF-motif for one point of contact, Sds22 has previously been identified to interact with PP1c through a larger structured region independent of the RVxF motif.^20,21^ Therefore, Sds22 binding to PP1c at the same time as mVenus-PDP1 in our data is consistent with the binding mode of Sds22.

**Fig. 3:**
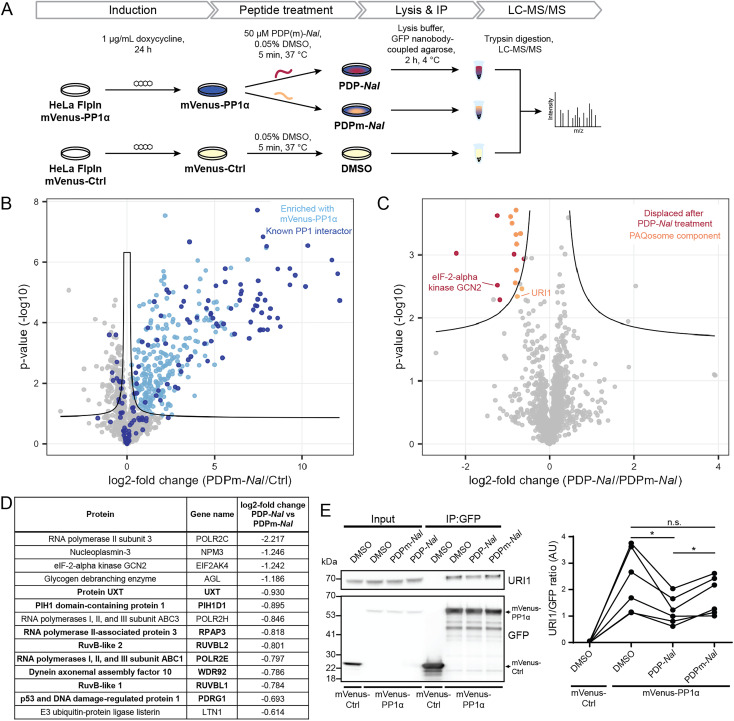
Determination of holoenzymes affected by PDPs using the cell-permeable PDP1 peptide versions PDP-NaI and PDPm-NaI. (A) Workflow to assess the impact of PDP-*Nal* on PP1 holoenzymes in intact cells using immunoprecipitation. (B) Results of MS experiment shown in A, comparing proteins bound to mVenus-PP1α treated with PDPm-NaI and mVenus-Ctrl treated with DMSO. Proteins significantly enriched with mVenus-PP1α compared to mVenus-Ctrl are highlighted in light blue, literature-known interactors are highlighted in dark blue. Significance threshold: FDR = 0.05, s0 = 0.1. (C) Results of MS experiment shown in A, comparing proteins bound to mVenus-PP1α after PDP-*Nal* treatment and PDPm-*Nal* treatment. Proteins significantly less abundant after PDP-*Nal* treatment compared to PDPm-*Nal* treatment are highlighted in red, components of the PAQosome protein complex are highlighted in orange. Proteins with a literature-known direct interaction with PP1 are labeled. Proteins that are significantly different but were not significantly enriched with mVenus-PP1α compared to mVenus-Ctrl in B are not highlighted. Significance threshold: FDR = 0.05, s0 = 0.1. (D) List of significantly different proteins from C that were also significantly enriched with mVenus-PP1α compared to mVenus-Ctrl in B, sorted by fold change. PAQosome components in bold. (E) Representative immunoblots and quantification of the experiment shown in A to assess changes in binding of URI1 to mVenus-PP1α. *n* = 6 biological replicates, Tukey multiple comparisons test, *p* values are multiplicity-adjusted. n.s. adjusted *p*-value > 0.05, * adjusted *p*-value < 0.05.

The comparison between PDP1 and PDP1m showed remarkable selectivity for PP1c for PDP1, highlighting the specificity of the RVTF motif. We next analyzed the data with regards to potential binding partners that bind the peptides outside of this motif. Since PDP1 and PDP1m are identical in those regions, any potential binding partner should also be common to both peptides and would not appear different in a comparison of the two. First, to separate proteins that bind to mVenus and those that may bind to PDPs irrespective of the RVTF or RATA sequence, we compared mVenus-PDP1(m) to mVenus-control pulldowns using the same anti-GFP beads and immunoprecipitation conditions (Fig. 3A). We also cross-checked our data against literature data for proteins unspecifically enriched in affinity purification MS experiments from HeLa cells.^19^ When considering all 1054 proteins identified in all replicates of at least one condition in the pulldown experiment (ESI Table 1†), 495 were also found in the mVenus-control pulldowns and are therefore considered as not specific to PDPs. We also excluded bait mVenus-PDP1(m) and the GFP nanobody. Of the remaining 557 proteins, 182 are listed in the CRAPome database^19^ as background contaminants, leaving 375 proteins that are potentially interacting with PDPs based on amino acid sequences irrespective of the intact RVTF motif. For mVenus-PDP1, 193 proteins were found; for mVenus-PDP1m, 372 proteins were found, and 190 proteins were common to both. The majority of these proteins showed a low MS intensity (Fig. 2C), proteins indicated in black; only three proteins with unique peptide matches were among the 100 most intense proteins found for mVenus-PDP1 (Fig. 2C; SPT16, hnRNP C1/C2 and ERP44, encoded by SUPT16H, HNRNPC and ERP44, respectively, ESI Table 1†) when comparing the summed MS intensities across all replicates. One further intense potential off-target protein was identified as a “protein group”, for which only shared peptides between five proteins were identified (histone H2A types 1-C, 3, 1-B/E, 1-A and Histone H2AX, encoded by HIST1H2AC, HIST3H2A, HIST1H2AB, HIST1H2AA and H2AFX, respectively). The observed MS intensity is therefore the sum of all five possible proteins. Although it is possible that the intensity results from just one highly abundant protein, it is not unlikely that multiple proteins contribute and the abundance of each contributor is lower. Similarly, for PDP1m, three proteins that potentially bind PDP1 and PDP1m were among the 100 most abundant proteins (ESI Fig. S1 and Table 1†). While it is encouraging that proteins that potentially bind PDPs outside the RVTF motif tended to be less intense hits, their presence nonetheless highlights the benefit of using a pair of compounds that ideally only differ in activity on the desired target but have identical effects otherwise,^22^ such as PDP1 and PDP1m.

This experiment confirms the previously reported selectivity of PDPs for PP1c over PP2A and Calcineurin, and reveals a remarkable specificity of the PDP RVTF sequence for PP1c across the human proteome. In a cellular context, little off-target binding is observed and, importantly, binding to other proteins is common to both PDP1 and PDP1m. This pair of peptides, combined with a general vehicle control, is therefore suitable for the study of the effect of PP1 holoenzyme disruption and the resulting effects on the phosphoproteome.

### 2.2 Treatment of cells expressing mVenus-PP1α with PDP-*Nal* leads to disruption of specific PP1 holoenzymes

Having established the selectivity of PDP1 for the catalytic subunit of PP1, we aimed to identify how PDPs affect holoenzymes of PP1. As previous studies had shown that PP1-dependent calcium oscillations could be observed within five minutes after cellular PDP treatment,^14^ we opted for this timeframe to study which RIPPOs (PPP1Rs) are most affected by PDPs. In this experiment, the cell-permeable peptide PDP-*Nal*^12^ (RRKRPKRKRKNARVTF*Nal*EAAEII, *Nal* = 2-naphthylalanine) and its inactive analogue PDPm-*Nal* (RRKRPKRKRKNARATA*Nal*EAAEII) were used.

To enable the isolation of PP1 holoenzymes, we used a fusion protein consisting of the α-isoform of PP1c, PP1α, and the fluorescent protein mVenus. This fusion protein forms holoenzymes with interactors of PP1 that could then be disrupted by PDPs. To stably express the mVenus-PP1α fusion protein or a control construct mVenus-Ctrl, an inducible HeLa Kyoto cell line was generated. Tagging of PP1α at the N-terminus and the presence of a linker was used to prevent interference with the normal function of PP1α.^23^ The control construct mVenus-Ctrl also included the linker section so that the only difference between mVenus-PP1α and mVenus-Ctrl is the sequence of PP1α. The HeLa Kyoto FlpInTrex mVenus-PP1α/Ctrl cells were induced with doxycycline 24 hours before PDP treatment. The medium was then exchanged for medium containing 50 μM PDP-*Nal*, 50 μM PDPm-*Nal* or the corresponding dimethyl sulfoxide (DMSO) concentration for the control construct, and after 5 minutes incubation at 37 °C, cells were lysed, and co-immunoprecipitation (Co-IP) of complexes using GFP-Trap beads and label-free quantitative mass spectrometry analysis were carried out (Fig. 3A).

Initially, we confirmed the expression of the constructs. While both proteins were expressed successfully, the expression efficiency of mVenus-PP1α was notably lower compared to mVenus-Ctrl during initial transient test transfection, as well as after the generation of an inducible stable cell line (ESI Fig. S2A and B†). In addition, the amount of endogenous PP1c was reduced in response to overexpression of the fusion protein mVenus-PP1α (ESI Fig. S2B†). This known effect^24^ is linked to the importance of PP1c activity for homeostasis and the intrinsic cytotoxicity of PP1α overexpression. Therefore, the expression efficiency could not be optimized further, and the difference in expression levels remained beyond what could be normalized at later stages of the experiment during Co-IP or MS analysis. However, the study setup was not negatively affected by this effect for two reasons: (1) the mVenus-Ctrl construct serves the purpose to identify proteins not binding PP1α but mVenus and the linker. More bait protein and therefore higher signal of mVenus-Ctrl and its bound proteins compared to mVenus-PP1α makes the setup even more robust to detect these proteins when analyzing differential binding between mVenus-PP1α and mVenus-Ctrl. (2) the core biological question of PP1 holoenzyme complexes dissociating upon PDP-*Nal* treatment is answered by comparing PDP-*Nal* and PDPm-*Nal* treatment of mVenus-PP1α. For both of these conditions, the same cell line was used with identical expression levels.

We next investigated the ability of mVenus-PP1α to form holoenzymes by comparing the proteins isolated in the Co-IP experiment. The formation of holoenzymes by the mVenus-PP1α fusion protein was assessed by comparing MS intensities of proteins bound to mVenus-PP1α after treatment with the inactive peptide PDPm-*Nal* to those bound to mVenus-Ctrl. A two-sided unpaired student's *t*-test between the two conditions revealed 458 significantly different proteins (threshold: FDR = 0.05, s0 = 0.1), of which 318 were enriched for mVenus-PP1α (Fig. 3B and ESI Table 2†). We compared these enriched proteins to proteins annotated as PPP1R in the HGNC database^25^ as well as those reported to bind PP1 through an RVxF motif in the KVxF PP1 docking motif repository^26^ and interactors of PP1α reported in DEPOD.^10^ Of 146 known interactors of PP1α found by MS in this experiment, 83 were enriched with mVenus-PP1α (indicated in dark blue in Fig. 3B). The fifteen most enriched proteins with mVenus-PP1α compared to mVenus-Ctrl are all known interactors of PP1c, as are 41 of the 50 most enriched proteins. This shows a strong capture of PP1-interacting proteins by mVenus-PP1α.

The analysis also revealed that 140 proteins were enriched more with mVenus-Ctrl. However, only nine known PP1 interactors behaved in this manner and the fold change for these proteins was generally smaller than in the proteins enriched with mVenus-PP1α (Fig. 3B). The apparent preferential binding to mVenus-Ctrl of some proteins was possibly the result of the difference in expression levels. Nonetheless, our experiment illustrates the successful formation of PP1 holoenzymes by the mVenus-PP1α fusion protein after 24 hours expression and shows that binding is maintained throughout the immunoprecipitation workflow. This experimental set-up is therefore suitable to study the effects of PDP-*Nal* on PP1 holoenzymes.

An analogous analysis of the MS data was carried out to compare the changes to holoenzymes after PDP-*Nal* treatment to control PDPm-*Nal* treatment. Using a two-sided unpaired Student's *t*-test to compare mVenus-PP1α treated with either PDP-*Nal* or PDPm-*Nal*, we found that only 17 proteins differed significantly (threshold: FDR = 0.05, s0 = 0.1, Fig. 3C and ESI Table 2†). For our analysis, we excluded three proteins that were not enriched with mVenus-PP1α *versus* mVenus-Ctrl in the previous experiment described before. The remaining fourteen proteins (Fig. 3D) showed reduced binding to mVenus-PP1α after PDP-*Nal* treatment compared to inactive PDPm-*Nal*, and are therefore candidates for interactors of PP1c that are susceptible to displacement by PDP-*Nal*. Interestingly, eight of these fourteen proteins are members of a protein complex called Particle for Arrangement of Quaternary Structure (PAQosome), including the proteins PDRG1 (p53 and DNA damage-regulated protein 1), PIH1D1 (PIH1 domain-containing protein 1), POLR2E (RNA polymerases I, II, and III subunit ABC1), RPAP3 (RNA polymerase II-associated protein 3), RuvB-like 1, RuvB-like 2, Protein UXT and WDR92 (Dynein axonemal assembly factor 10). The PAQosome, formerly known as the R2TP/Prefoldin-like complex, is a chaperone complex composed of twelve proteins,^27^ ten of which are part of two multisubunit modules.^28^ A further two proteins that are part of the PAQosome (PFDN2 and URI1) were also displaced from mVenus-PP1α in this experiment, although slightly below the significance threshold. It is notable that all ten PAQosome components identified in this experiment differed by a similar fold-change (Fig. 3D, log 2 fold change between −0.930 and −0.664), consistent with their association in one protein complex.

One of the ten identified PAQosome components found in this experiment has a known direct association with the catalytic subunit of PP1: the complex component unconventional prefoldin RPB5 interactor 1 (URI1), was previously identified as an oncogene^29^ and named as PP1 regulatory subunit 19 (PPP1R19).^30^ URI1 was shown to be the point of interaction between the PAQosome and PP1c.^27^ Disruption of the interaction between URI1 and PP1c leads to the dissociation of the entire PAQosome complex from PP1c,^27^ as also observed in our data. The finding of the PAQosome complex being a target after this short PDP treatment is especially intriguing when taking the short linear motif (SLiM) RVxF of URI1 with PP1c into account: The RVxF consensus motif for PP1c binding is RVEF in case of human URI1.^31^ The negative charge of glutamic acid (E) is known to make the motif particularly weak in terms of affinity to the catalytic subunit and can therefore be competed away easily by PDP-*Nal*.

As URI1 is the known point of interaction between the PAQosome and PP1c, we sought to confirm the displacement of URI1 from mVenus-PP1α by PDP-*Nal* during the 5 minutes treatment of HeLa FlpInTrex cells using a different method. The Co-IP experiment was repeated and the eluates were analyzed for URI1 by immunoblotting. Although the difference in the amount of URI1 bound to mVenus-PP1α compared to the control was slightly below the significance threshold when observed by MS (Fig. 3C), in this immunoblotting experiment the relative amount of URI1 bound to mVenus-PP1α decreased significantly after PDP-*Nal* treatment compared to PDPm-*Nal* treatment (Fig. 3E and ESI Fig. 2C†), confirming the disruption of the PP1c–URI1 interaction by PDP-*Nal* after only 5 minutes treatment.

Another interaction that was disrupted by PDP-*Nal* within 5 minutes is the complex of mVenus-PP1α with eIF-2-alpha kinase GCN2 (encoded by EIF2AK4). eIF-2-alpha kinase GCN2 was also previously identified as an interactor of PP1c^32,33^ and contains two potential RVxF motifs, RVRF and RILF, although the site of interaction has not been determined. The remaining five significantly different proteins, RNA polymerase II subunit 3, nucleoplasmin-3, glycogen debranching enzyme, RNA polymerases I, II, and III subunit ABC3, and E3 ubiquitin-protein ligase listerin (Fig. 3D) have no known association with PP1c yet. Since these proteins and their interaction with PP1 appear to be dynamic, they are promising candidates for further studies regarding their role in cellular signaling by PP1.

Collectively, these results provide evidence for the direct targeting of PP1 holoenzymes by PDP-*Nal* in intact human cells already after 5 minutes. Certain holoenzymes, such as the complex between PP1 and URI1 and between PP1 and eIF-2-alpha kinase GCN2, appear to be more susceptible targets of PDP-*Nal*, with dissociation just five minutes after adding the peptide to the growth medium.

### 2.3 PDPs lead to shifts in the kinase/phosphatase equilibrium for PP1-holoenzyme substrate sites

**Fig. 4:**
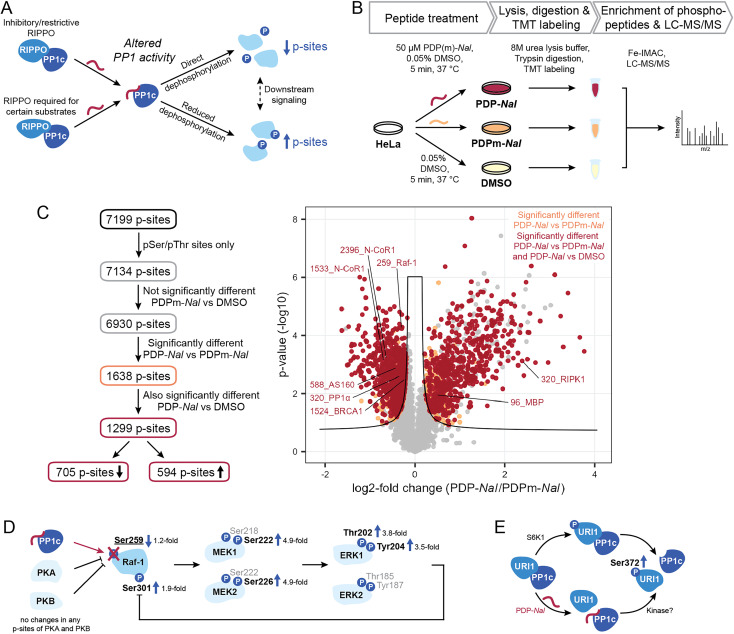
Determination of phosphorylation sites and pathways affected by PDPs. (A) Different effects of PDP-*Nal* modulating PP1 activity for different types of RIPPOs and substrates. (B) Workflow to assess the impact of PDP-*Nal* on the phosphoproteome. (C) Filtering steps and results of phosphoproteomic experiment shown in B, comparing pSer/pThr phosphosites after PDP-*Nal* and PDPm-*Nal* treatment. pSer/pThr phosphosites significantly different after PDP-*Nal* treatment compared to PDPm-*Nal* treatment are highlighted in orange, pSer/pThr phosphosites significantly different after PDP-*Nal* treatment compared to both PDPm-*Nal* treatment and DMSO treatment are highlighted in red. Sites above the line of significance in grey differed significantly between the two controls PDPm-*Nal* and DMSO. Selected known PP1 substrate sites are labeled. Significance threshold: FDR = 0.05, s0 = 0.1. (D) MAP Kinase signaling pathway initiated by activation of Raf-1. Red = dephosphorylation, black = phosphorylation, site pSer259 in bold and underlined was significantly decreased after PDP-*Nal* treatment compared to PDPm-*Nal* treatment, sites in bold were significantly increased, sites in grey were not observed. (E) Mechanisms leading to dissociation of URI1 from PP1 and phosphorylation at Ser372. Site in bold was significantly increased.

Having shown the selectivity of PDPs and identified holoenzymes disrupted after 5 minutes of PDP treatment, we sought to investigate what effects PDP treatment has on the phosphoproteome in the same time frame. The disruption of PP1 holoenzymes is expected to have different consequences depending on the function of the RIPPO that is being removed (Fig. 4A). Many RIPPOs have an inhibitory or restrictive effect on PP1 activity;^30^ their replacement by PDPs should therefore lead to increased activity on proximal substrates of the PDP-PP1 complex and thus reduce phosphorylation levels of these substrates. Conversely, RIPPOs may be required for dephosphorylation of certain substrates, and in these cases loss of the RIPPO triggered by PDPs would lead to reduced activity on the substrate and therefore increased phosphorylation levels. However, increased phosphorylation may also be the consequence signaling cascades resulting in the activation of kinases.

To measure the global phosphoproteome changes, we replaced the medium of HeLa Kyoto cells with medium containing either 50 μM PDP-*Nal*, PDPm-*Nal* or a DMSO control solution and incubated them for five minutes at 37 °C. The use of two controls (PDPm-*Nal* and DMSO) provided a more stringent quality control. Following incubation, cells were immediately placed on ice and lysed in 8 M urea. Samples were TMT-labeled and MS analysis was performed (Fig. 4B). The dataset was normalized for the median of total TMT reporter intensities across all samples. After filtering only for class I phosphorylation sites, *i.e.* phosphorylation sites with a localization probability of >0.75, the dataset consisted of 7199 p-sites (ESI Table 3†). As our focus were sites that are directly controlled by PP1, 65 pTyr sites were excluded since they are likely the result of downstream signaling rather than direct dephosphorylation by the Ser/Thr-specific phosphatase PP1. The remaining 7134 p-sites were analyzed further (Fig. 4C).

Of all 7134 pSer/pThr sites, we first excluded 204 p-sites that differed significantly between the two control treatments, PDPm-*Nal* treatment and DMSO treatment, as they changed even in the absence of PP1 modulation (ESI Fig. S3†). Of the remaining 6930 p-sites, 1638 p-sites differed significantly between PDP-*Nal*-treated and PDPm-*Nal*-treated samples in a two-sided unpaired Student's *t*-test (Fig. 4C). 1,299, or 79% of these 1638 candidate sites were also significantly different when comparing PDP-*Nal* to the DMSO control (Fig. 4C and ESI Table 3†). Further analysis of the 1299 p-sites showed that 705 sites are less phosphorylated following PDP-*Nal* treatment compared to PDPm-*Nal* treatment, while 594 p-sites increased in phosphorylation. These sites encompass direct substrates of PP1 that are experiencing altered dephosphorylation as a result of the PDP treatment, but also include indirect effects resulting from signaling cascades such as activation of kinases or other phosphatases. Therefore, this resulting dataset was then compared against databases of PP1-regulated phosphorylation sites and PP1 substrates or datasets from related experiments.

We searched sites affected by PDP-*Nal* for sites known to be regulated by PP1 (ref. 10) and found five such sites, four of which showed decreased phosphorylation (Fig. 4C). The sites that decreased in phosphorylation are pThr320 of PP1α (gene PPP1CA), which undergoes auto-dephosphorylation,^11,34^ pSer259 of Raf-1 (gene RAF1),^35^ pSer1524 of BRCA1 (ref. 36) and pSer588 of AS160 (gene TBC1D4),^37^ while pSer320 of RIPK1 (ref. 38) increased after PDP-*Nal* treatment. Three further sites in our data are located on two proteins that are known PP1 substrates, MBP^39,40^ and N-CoR1 (ref. 41) (gene NCOR1), but the exact site under PP1 regulation is unknown. Of these sites, two exhibited reduced phosphorylation, while one was increased. Of note, the small number of known sites in our data set reflects the fact that, while PP1 is a ubiquitous phosphatase, only few substrate sites have been annotated so far (<100),^10^ making this comparison challenging.

Dephosphorylation of pSer259 on Raf-1 by PP1 leads to activation of Raf-1 and the Raf1-MEK-ERK cascade.^35^ This cascade involves Raf-1 phosphorylating the kinases MEK1 (gene MAP2K1) and MEK2 (gene MAP2K2) at Ser218 and Ser222 or Ser222 and Ser226, respectively,^42^ which in turn then phosphorylate ERK1 (gene MAPK3) and ERK2 (gene MAPK1) at Thr202 and Tyr204 or Thr185 and Tyr187, respectively.^43^ Activation of ERK1/2 then leads to phosphorylation of Raf-1 at Ser301 in a feedback loop.^44,45^ In our phosphoproteomics data, activation of this cascade by PDP-*Nal* was observed (Fig. 4D): phosphorylation of Raf-1 at pSer259 was decreased and phosphorylation of MEK1/2 at pSer222/pSer226, ERK1 at pThr202 and pTyr204, and Raf-1 at pSer301 was increased. For the kinases PKA^46^ (genes PRKACA, PRKACB and PRKACG) and PKB^47^ (gene AKT1) which are responsible for the inhibitory phosphorylation of Raf-1, no phosphopeptides with altered intensities were observed, so there is no indication for altered kinase activity upstream of Raf-1 as the cause of the change in pSer259 of Raf-1. Additionally, the amplification of signal is evident in the phosphorylation changes. The phosphorylation of pSer259 in Raf-1 was only decreased 1.2-fold, while the downstream signals of MEK1, MEK2 and ERK1 were increased approximately 3.5- to 4.9-fold (Fig. 4D). Thus, with this approach we could observe downstream signaling, which illustrates that the changes observed after 5 minutes PDP treatment already include indirect effects. In previous experiments using PDPs to interrogate MAPK signaling, dephosphorylation of MEK1/2 was observed after 20 minutes PDP-*Nal* treatment.^12^ This indicates that depending on the conditions, MEK1/2 phosphorylation is regulated either directly or indirectly by PP1.

The site pSer320 on RIPK1 is an inhibitory p-site that is dephosphorylated by the PP1γ-PPP1R3G holoenzyme, which is required for recruitment of PP1 to RIPK1.^38^ Since this RIPPO binds PP1c through an RVQF motif, treatment with PDP-*Nal* is expected to result in a loss of recruitment of PP1 to RIPK1 and therefore an increase in phosphorylation (Fig. 4A), which is what we observed in our data. The kinases responsible for pSer320 phosphorylation, MAP kinase-activated protein kinase 2 (ref. 48) (gene MAPKAPK2) and ULK1,^49^ are not significantly altered in phosphorylation and therefore unlikely to cause the change in phosphorylation after PDP-*Nal* treatment.

With our finding of the URI1-PP1c complex being a target of PDP-*Nal*, we explored whether we could observe changes in phosphorylation related to this complex dissociation. It was previously shown that the URI1-PP1c interaction is mainly regulated by the phosphorylation of Ser372 on URI1, a site phosphorylated by S6 kinase (S6K1) with an important role in cancer formation.^50^ Phosphorylation of URI1 at Ser372 by S6K1 results in the dissociation of URI1 from PP1γ, releasing PP1c (Fig. 4E). Interestingly, while dephosphorylation of URI1 by PP1c *in vitro* has been reported,^51^ association of PP1γ and URI1 could be prevented by phosphorylating URI1 prior to incubation with PP1c,^50^ indicating that PP1c dephosphorylates URI1 to induce complex formation only in certain conditions, as observed for high glucose levels or high PP1 expression levels.^51^ This correlation between increased pSer372 phosphorylation and decreased PP1c association was also apparent in our data (Fig. 4C), with the site significantly more phosphorylated in PDP-*Nal* treated cells compared to both, cells treated with PDPm-*Nal* or DMSO. However, unlike in previous work on this interaction, the disruption of the complex appears to have triggered the increase in phosphorylation rather than the opposite mechanism described in the literature (Fig. 4E). The downstream signaling that was described as a consequence of URI1 phosphorylation in mitochondria was not observed here, possibly due to the short time frame.^50^ The disruption of the URI1-PP1c complex is also of therapeutic interest, as URI1 was identified as an “addictive” oncogene in ovarian cancer where expression levels of URI1 are increased.^29^ A particularly interesting finding in the context of the URI1-PP1 complex is that URI1 was reported to enhance survival through sequestration of PP1c by binding it and keeping it inactive.^29^ Disruption of the complex could therefore release PP1c and reduce survival signaling.

### 2.4 Comparison of PDP *versus* PP1 catalytic subunit-derived phosphoproteomic data offers high confidence substrate candidates

**Fig. 5:**
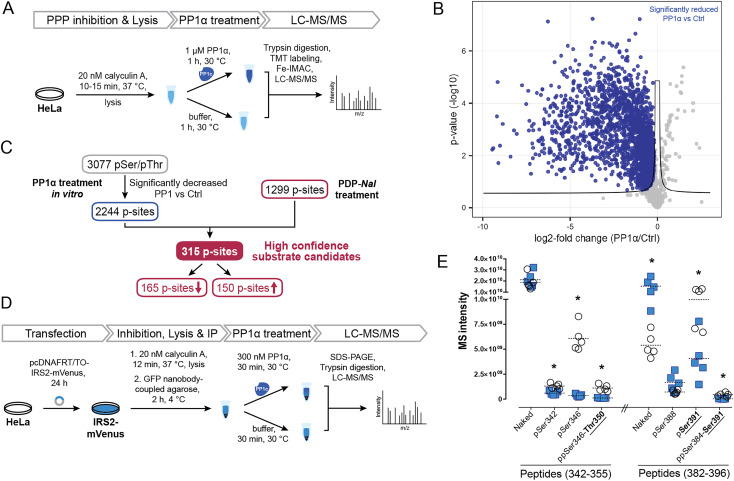
Determination of new high confidence substrates of PP1c. (A) Workflow of an *in vitro* dephosphorylation experiment^52^ using recombinant PP1α. (B) Results of MS experiment shown in A, comparing pSer/pThr phosphosites after PP1α treatment and untreated controls. pSer/pThr phosphosites significantly decreased after PP1c treatment compared to control highlighted in blue. Significance threshold: FDR = 0.05, s0 = 0.1. (C) Integration of *in vitro* experiment (Fig. 4A and B) with results after PDP-*Nal* treatment (Fig. 3B and C). (D) Workflow of *in vitro* dephosphorylation of IRS2-mVenus using recombinant PP1α. (E) Results of *in vitro* dephosphorylation of IRS2-mVenus showing significantly changed phosphosites on indicated IRS2 (phospho)peptides compared using a two-sided unpaired multiple *t*-test. * *p*-value < 0.05; *n* = 5.

To identify potential PP1 substrate sites and further detangle direct from indirect effects, we compared the sites that were affected by PDP-*Nal* treatment to a complementary experiment using recombinant PP1α catalytic subunit (Fig. 5A). In this previously reported experiment,^52^ cells were treated with the phosphoprotein phosphatase inhibitor calyculin A to inhibit endogenous phosphatases, lysed and then incubated with the catalytic subunit PP1α at 30 °C for 1 h. P-sites and changes in their phosphorylation status were identified using MS.

To compare changes in phosphorylation after the two experiments, our previously published dataset^52^ was reanalyzed using total intensities and filtered for pSer/pThr sites with a localization probability of >0.75. Comparing PP1α-treated lysates with untreated controls, 2449 significantly changed sites were identified, the majority of which, 2443, were pSer or pThr sites, consistent with the known substrate specificity of PP1 (ESI Table 3†). 2376 pSer/pThr sites decreased after PP1α treatment while 67 sites increased (Fig. 5B), in line with minimal downstream signaling occurring and direct dephosphorylation being the dominant event. Sites dephosphorylated in this experiment are highly likely to be direct substrates of PP1α; however, localization of proteins that may influence their susceptibility to dephosphorylation by PP1 is lost in this experiment. In addition, using recombinant PP1α at non-endogenous concentrations and for extended times may lead to dephosphorylation events that would not occur in the native environment of the cell. Then, by comparing the cellular treatment with PDPs (Fig. 4C), which maintains spatial information and affects all PP1 isoforms (PP1α,β,γ), with the in lysate dephosphorylation by recombinant PP1α (Fig. 5B), which minimizes indirect effects, we were able to determine 315 high confidence substrate candidates of recombinant PP1α and PDP-bound PP1c (Fig. 5C and ESI Table 3†). 165 of 315 of the p-sites that changed following PP1 modulation or treatment decreased in phosphorylation following PDP-*Nal* treatment, while 150 sites were more frequently phosphorylated. The known substrate sites on PP1α and Raf-1 are both among the set of sites that was dephosphorylated both in lysate and after PP1 modulation by PDP-*Nal*. URI1 on the other hand was found to be more phosphorylated after PDP-*Nal* treatment and was not dephosphorylated by recombinant PP1α in lysate, as expected for the site based on the literature data described above. Among the 150 sites that increased in phosphorylation after PDP-*Nal* treatment and were PP1α substrates in lysate, three sites are already known to be regulated by PP1: pSer320 on RIPK1, pSer25 of Stathmin (encoded by STMN1) and pSer222/226 of MEK1/2. As discussed above, the dephosphorylation of pSer320 on RIPK1 by PP1c is consistent with the literature.^38^ The pSer25 site of Stathmin is a known substrate of PP1c *in vitro*, but is also sensitive to PP2A and calcineurin^53^ and is phosphorylated by ERK1/2.^54^ As ERK1 is activated as a result of signaling following Raf-1 activation by PDP-*Nal* treatment (Fig. 4D), it is plausible that the observed increase of pSer25 of Stathmin results from ERK1 activity. The third site, pSer222/226 of MEK1/2, is a known substrate of PP1c^12^ but after short PDP-*Nal* treatment in cells phosphorylation by activated Raf-1 dominates, as discussed above.

Having established that the dephosphorylation of known PP1 substrates can be modulated by PDP-*Nal*, we further investigated a high confidence substrate candidate resulting from the above data comparison. Insulin receptor substrate-2 (IRS2) experienced significant changes in phosphorylation of ten residues after PDP-*Nal* treatment (ESI Table 3†); eight of these sites were less phosphorylated than in control conditions, and five of these residues were also dephosphorylated by PP1c *in vitro*,^52^ making these sites strong candidates for substrate sites of PP1. To date, no association between PP1 and IRS2 has been reported, although a protein complex containing PP1c, IRS1 and PPP1R12A has been found to play a role in insulin signaling.^55,56^ To assess PP1 regulation of IRS2 phosphorylation in a more targeted manner, we carried out an *in vitro* dephosphorylation assay of enriched IRS2 (Fig. 5D). To this end, a plasmid encoding an IRS2-mVenus fusion protein was generated and HeLa cells were transfected transiently. IRS2-mVenus was enriched using GFP-Trap beads and recombinant PP1α was added. Following incubation and elution, the fusion protein was isolated using SDS-PAGE, digested and analyzed using MS. We first used discovery driven proteomics and identified a large number of phosphosites on IRS2. Next, we manually validated those IRS2 phosphopeptides as well as corresponding not-phosphorylated peptide sequences using the targeted proteomics software Skyline.^57^ With this approach we could identify 12 phosphosites within the IRS2 protein that were dephosphorylated by PP1α (ESI Table 3†). MS intensities for two particularly interesting regions that were identified in the previous two experiments, amino acids 342–355 and 382–396, and their changes in phosphorylation are shown in Fig. 5E (ESI Fig. S4 and Table 3†). This finding confirms dephosphorylation events previously identified in similar regions of the IRS2 proteins, like Thr350 and Ser391 (Fig. 5E); while other regions affected by PDP-*Nal* containing sites 520, 527 and 620 were not found to be dephosphorylated in this assay. This may be due to different phosphorylation patterns caused by the presence of a tag, or overexpression, or steric inaccessibility of the regions once bound to beads. Interestingly, for some phosphosites we could detect an increase in the not-phosphorylated peptide version as it shown for naked peptide (382–396) in Fig. 5E (3.5-fold upregulation upon phosphatase treatment). This suggests that the phosphorylation site occupancy at these sites before the treatment was rather high. In summary, our experiment shows that IRS2 is a direct target of PP1c and gets dephosphorylated at several biologically relevant sites.

## 3 Conclusion

We developed here a MS-based strategy that aimed at disentangling direct *versus* indirect effects of an enzyme modulator, namely PDP-*Nal*. After determining the exquisite selectivity of the PDP for PP1, we found that PDPs do not affect all holoenzymes equally, and identified those few that were most affected after a short 5 minutes treatment. The susceptibility of the interaction between URI1 and PP1c to disruption by PDP-*Nal* is of particular interest for further studies, as this interaction plays a role in survival signaling in liver cancer.^51^ At the same time, this 5 minutes PDP treatment of cells already led to remarkable changes in the phosphoproteome, which needs to be considered in their application as modulators of PP1 and as PhoRCs. Many changes were the direct result of dephosphorylation by PP1, such as the observed dephosphorylation of PP1 itself and Raf-1. Other sites change as a result of rapid signaling, such as MEK1/2 and ERK1/2, which can be visualized by the approaches developed here. Importantly, by combining this dataset with data from in lysate experiments and literature data, we begin to separate these effects and identify new PP1 substrate candidates, such as IRS2. On the other hand, the data provides clues as to which PP1 substrates require RIPPOs for dephosphorylation and are not dephosphorylated by the catalytic subunit alone in cells, such as RIPK1. Together, this illustrates that disrupting only a subset of holoenzymes is a plausible avenue to further detangle the complex network of PP1 regulation and provides here a rich dataset on potential substrates of PP1. Due to the general applicability of the MS read-out, we expect that this strategy will be transferable to determine the action of modulators of other enzymes. Our strategy provides a way to determine whether observed effects are due to the desired target of a chemical modulator. This offers an alternative to the recommended strategy^58^ of using a chemically distinct modulator that affects the same enzyme, since such orthogonal probes are not available for many targets.

## Data availability

All data and methods are available in the manuscript or the ESI.† The mass spectrometric raw files as well as the MaxQuant output files have been deposited to the ProteomeXchange Consortium *via* the PRIDE partner repository and can be accessed using the identifier PXD044415 (https://proteomecentral.proteomexchange.org/cgi/GetDataset?ID=PXD044415). The mass spectrometric raw files analyzed with the Skyline software have been deposited to Panorama Public^59^ and can be accessed *via*https://panoramaweb.org/PP1_PDPs.url.

## Author contributions

B. H. and E. M. D. contributed equally. B. H. designed, optimized and performed the MS experiments using PDPs. E. M. D. performed immunoblotting experiments. M. E. performed the MS experiment with IRS2. C. L. carried out LC-MS/MS measurements. B. H., E. M. D., M. E., M. K. and C. L. analyzed the data. E. M. D., B. H. and M. K. wrote the manuscript. M. K. conceived, designed and supervised the study. All authors edited the manuscript.

## Conflicts of interest

There are no conflicts to declare.

## Supplementary Material

SC-015-D3SC04746F-s001

SC-015-D3SC04746F-s002
