# Supplementary material for: A strategy to disentangle direct and indirect effects on (de)phosphorylation by chemical modulators of the phosphatase PP1 in complex cellular contexts
Source: Chem Sci. 2024 Jan 12;15(8):2792–804. doi: 10.1039/d3sc04746f (PMC10882499; doi:10.1039/d3sc04746f)
Supplement: SC-015-D3SC04746F-s001 [file SC-015-D3SC04746F-s001.pdf]

Electronic Supplementary Material (ESI) for Chemical Science.
This journal is © The Royal Society of Chemistry 2024

Electronic Supplementary Information

A strategy to disentangle direct and indirect effects on (de)phosphorylation by chemical modulators of the phosphatase PP1 in complex cellular contexts

Bernhard Hoermann[a,b,e], Eva-Maria Dürr[a,b,e], Christina Ludwig[c,d], Melda Ercan[a,b], Maja Köhn[a,b,*]

[a] Faculty of Biology, Institute of Biology III, University of Freiburg, Freiburg, Germany

[b] Signalling Research Centres BIOSS and CIBSS, University of Freiburg, Freiburg, Germany

[c] Chair of Proteomics and Bioanalytics, Technical University of Munich (TUM), Freising, Germany.

[d] Bavarian Center for Biomolecular Mass Spectrometry (BayBioMS), Technical University of Munich (TUM), Freising, Germany.

[e] These authors contributed equally.

* Correspondence to: maja.koehn@bioss.uni-freiburg.de

Contents

1. Experimental methods 3
1.1. Cloning 3
1.1.1. Cloning of pTriEx-mVenus-PDP1 and pTriEx-mVenus-PDP1m 3
1.1.2. Cloning of pcDNA-FRT/TO-mVenus-PP1α or –Ctrl 4
1.1.3. Cloning of pcDNA-FRT/TO-IRS2-mVenus 6
1.2. Cell Culture, transfection and generation of stable cell lines 7
1.3. Cell Culture Experiments 8
1.3.1. Co-immunoprecipitation experiment with mVenus-PDP1(m) 8
1.3.2. Co-immunoprecipitation experiment with mVenus-PP1α after PDP(m)-*Nal* treatment 9
1.3.3. PDP(m)-*Nal* treatment and sample preparation for phosphoproteomics 12
1.3.4. Recombinant PP1α treatment on immunoprecipitated IRS2-mVenus and sample preparation for phosphoproteomics 14
1.4. Data analysis and integration of in lysate dephosphorylation experiment reported previously 16
2. Supplementary figures 17
3. Raw images of Western blots 20
4. Description of additional supplementary materials 24
5. References 25

Other Supplementary Materials for this manuscript include the following:

Supplementary Tables 1-3

1. Experimental methods

1.1. Cloning

All PCRs were carried out using Phusion Polymerase (Thermo Fisher), digestions were performed using FastDigest enzymes and buffer (Thermo Fisher).

1.1.1. Cloning of pTriEx-mVenus-PDP1 and pTriEx-mVenus-PDP1m

pTri-Ex-mVenus-PDP1 was a gift from the Hahn Lab (University of North Carolina, Chapel Hills). The RFTV-binding motif in PDP1 was mutated to RATA using a two-step approach. In a first PCR round, two fragments were created from pTriEx-mVenus-PDP1 using primer pairs AACGCTCGCGCCACTGCCGCCGAA (fw) and GCTCAAGGGGCTTCATGATGTCC (rev) and CAATCAAAGGAGATATACCATGGTGAGCAAGG (fw) and TTCGGCGGCAGTGGCGCGAGCGTT (rev). The resulting overlapping fragments containing the mutated RATA sequence were purified on a 0.8% agarose gel and used together as template in the second step PCR with the flanking primers CAATCAAAGGAGATATACCATGGTGAGCAAGG (fw) and GCTCAAGGGGCTTCATGATGTCC (rev). The resulting fragment as well as the original pTriEx-mVenus –PDP1 plasmid were then both digested using NcoI/HindIII and the final PCR fragment was inserted. The integrity of the newly generated plasmid was verified using Sanger sequencing.

DNA sequence encoding mVenus-PDP1 with NcoI and HindIII restriction sites used for restriction digest and ligation into pTriEx vector:

CAATCAAAGGAGATATACCATGGTGAGCAAGGGCGAGGAGCTGTTCACCGGGGTGGTGCCCATCCTGGTCG
AGCTGGACGGCGACGTAAACGGCCACAAGTTCAGCGTGTCCGGCGAGGGCGAGGGCGATGCCACCTACGGC
AAGCTGACCCTGAAGTTCATCAGCACCACCGGCAAGCTGCCCGTGCCCTGGCCCACCCTCGTGACCACCCTCG
GCTACGGCCTGCAGTGCTTCGCCCGCTACCCCGACCACATGAAGCAGCACGACTTCTTCAAGTCCGCCATGCC
CGAAGGCTACGTCCAGGAGCGCACCATCTTCTTCAAGGACGACGGCAACTACAAGACCCGCGCCGAGGTGAA
GTTCGAGGGCGACACCCTGGTGAACCGCATCGAGCTGAAGGGCATCGACTTCAAGGAGGACGGCAACATCCT
GGGGCACAAGCTGGAGTACAACTACAACAGCCACAACGTCTATATCACCGCCGACAAGCAGAAGAACGGCAT
CAAGGCAAACTTCAAGATCCGCCACAACATCGAGGACGGCGGCGTGCAGCTCGCCGACCACTACCAGCAGAA
CACCCCCATCGGCGACGGCCCCGTGCTGCTGCCCGACAACCACTACCTGAGCTACCAGTCCAAACTGAGCAAA
GACCCCAACGAGAAGCGCGATCACATGGTCCTGCTGGAGTTCGTGACCGCCGCCGGGATCACTCTCGGCATG
GACGAGCTGTACAAGGGATCCCGCCCAAAAAGAAAAAGAAAGAACGCTCGCGTGACTTTTGCCGAAGCCGCG
GAAATTATCTAAAAGCTTGCGGCCGCACAGCTGTATACACGTGCAAGCCAGCCAGAACTCGCCCCGGAAGAC
CCCGAGGATCTCGAGCACCACCATCACCATCACCATCACTAAGTGATTAACCTCAGGTGCAGGCTGCCTATCA
GAAGGTGGTGGCTGGTGTGGCCAATGCCCTGGCTCACAAATACCACTGAGATCGATCTTTTTCCCTCTGCCAA
AAATTATGGGGACATCATGAAGCCCCTTGAGC

DNA sequence encoding mVenus-PDP1m with NcoI and HindIII restriction sites used for restriction digest and ligation into pTriEx vector:

CAATCAAAGGAGATATACCATGGTGAGCAAGGGCGAGGAGCTGTTCACCGGGGTGGTGCCCATCCTGGTCG
AGCTGGACGGCGACGTAAACGGCCACAAGTTCAGCGTGTCCGGCGAGGGCGAGGGCGATGCCACCTACGGC
AAGCTGACCCTGAAGTTCATCAGCACCACCGGCAAGCTGCCCGTGCCCTGGCCCACCCTCGTGACCACCCTCG
GCTACGGCCTGCAGTGCTTCGCCCGCTACCCCGACCACATGAAGCAGCACGACTTCTTCAAGTCCGCCATGCC
CGAAGGCTACGTCCAGGAGCGCACCATCTTCTTCAAGGACGACGGCAACTACAAGACCCGCGCCGAGGTGAA
GTTCGAGGGCGACACCCTGGTGAACCGCATCGAGCTGAAGGGCATCGACTTCAAGGAGGACGGCAACATCCT
GGGGCACAAGCTGGAGTACAACTACAACAGCCACAACGTCTATATCACCGCCGACAAGCAGAAGAACGGCAT
CAAGGCAAACTTCAAGATCCGCCACAACATCGAGGACGGCGGCGTGCAGCTCGCCGACCACTACCAGCAGAA
CACCCCCATCGGCGACGGCCCCGTGCTGCTGCCCGACAACCACTACCTGAGCTACCAGTCCAAACTGAGCAAA

GACCCCAACGAGAAGCGCGATCACATGGTCCTGCTGGAGTTCGTGACCGCCGCCGGGATCACTCTCGGCATG
GACGAGCTGTACAAGGGATCCCGCCCAAAAAGAAAAAGAAAGAACGCTCGCGCCACTGCCGCCGAAGCCGC
GGAAATTATCTAAAAGCTTGCGGCCGCACAGCTGTATACACGTGCAAGCCAGCCAGAACTCGCCCCGGAAGA
CCCCGAGGATCTCGAGCACCACCATCACCATCACCATCACTAAGTGATTAACCTCAGGTGCAGGCTGCCTATCA
GAAGGTGGTGGCTGGTGTGGCCAATGCCCTGGCTCACAAATACCACTGAGATCGATCTTTTTCCCTCTGCCAA
AAATTATGGGGACATCATGAAGCCCCTTGAGC

Amino acid sequence of mVenus-PDP1:
MVSKGEELFTGVVPILVELDGDVNGHKFSVSGEGEGDATYGKLTLKFISTTGKLPVPWPTLVTTLGYGLQCFARYPD
HMKQHDFFKSAMPEGYVQERTIFFKDDGNYKTRAEVKFEGDTLVNRIELKGIDFKEDGNILGHKLEYNYNSHNVYI
TADKQKNGIKANFKIRHNIEDGGVQLADHYQQNTPIGDGPVLLPDNHYLSYQSKLSKDPNEKRDHMVLLEFVTAA
GITLGMDELYKGSRPKRKRKNARVTFAEAAEII

Amino acid sequence of mVenus-PDP1m:
MVSKGEELFTGVVPILVELDGDVNGHKFSVSGEGEGDATYGKLTLKFISTTGKLPVPWPTLVTTLGYGLQCFARYPD
HMKQHDFFKSAMPEGYVQERTIFFKDDGNYKTRAEVKFEGDTLVNRIELKGIDFKEDGNILGHKLEYNYNSHNVYI
TADKQKNGIKANFKIRHNIEDGGVQLADHYQQNTPIGDGPVLLPDNHYLSYQSKLSKDPNEKRDHMVLLEFVTAA
GITLGMDELYKGSRPKRKRKNARATAAEAAEII

1.1.2. Cloning of pcDNA-FRT/TO-mVenus-PP1α or –Ctrl

First a linker was added between mVenus and PP1α of an existing mVenus-PP1α construct. The gene PPP1CA encoding PP1α was obtained using pTriEx-mVenus-PPP1CA as a template and primers ATGTCCGACAGCGAGAAGC (fw) and CGCAAGCTTTTACTATTTCTTGGC (rev). The resulting PCR product was used as template in a second PCR with the forward primer ATTATTGGATCCGGCGGAAGCGGCTCCATGTCCGACAGCGAGAAGC and the same reverse primer as in the first PCR, with the aim to add an elongated C-terminal linker to PP1α. The resulting fragment was digested with BamHI and HindIII and ligated back into the BamHI/HindIII-digested vector of origin. In a second step, this plasmid was then used as template in a PCR with primers ATGGTGAGCAAGGGCGAGG (fw) and TTACTATTTCTTGGCTTTGGCGG (rev) to obtain mVenus-PP1α connected by the longer linker. The resulting fragment was used together with primers GTACAAGCTTGCCACCATGGTGAGCAAGGGCGAGG (fw) and CTTAGCGGCCGCTTTACTATTTCTTGGCTTTG-GCGG (rev) to attach HindIII and NotI sites, digested with HindIII and NotI, and ligated into a HindIII/NotI-digested pcDNA/FRT/TO entry vector. For the control containing mVenus and the linker but without PP1α, the same mVenus-PP1α fragment was used as a template and HindIII and NotI sites were added using the same forward primer as for mVenus-PP1α and reverse primer CTTAGCGGCCGCTTTACTAGGAGCCGCTTCCGCCG. The PCR product was digested with HindIII and NotI, and ligated into a HindIII/NotI-digested pcDNA/FRT/TO entry vector. All PCRs described herein were run on a 0.8% agarose gel for analysis and all plasmids were verified in terms of sequence integrity using Sanger sequencing.

DNA sequence encoding mVenus-PP1α with HindIII and NotI restriction sites used for restriction digest and ligation into pcDNA/FRT/TO entry vector:
GTACAAGCTTGCCACCATGGTGAGCAAGGGCGAGGAGCTGTTCACCGGGGTGGTGCCCATCCTGGTCGAGCT
GGACGGCGACGTAAACGGCCACAAGTTCAGCGTGTCCGGCGAGGGCGAGGGCGATGCCACCTACGGCAAGC
TGACCCTGAAGTTCATCAGCACCACCGGCAAGCTGCCCGTGCCCTGGCCCACCCTCGTGACCACCCTCGGCTA
CGGCCTGCAGTGCTTCGCCCGCTACCCCGACCACATGAAGCAGCACGACTTCTTCAAGTCCGCCATGCCCGAA
GGCTACGTCCAGGAGCGCACCATCTTCTTCAAGGACGACGGCAACTACAAGACCCGCGCCGAGGTGAAGTTC
GAGGGCGACACCCTGGTGAACCGCATCGAGCTGAAGGGCATCGACTTCAAGGAGGACGGCAACATCCTGGG
GCACAAGCTGGAGTACAACTACAACAGCCACAACGTCTATATCACCGCCGACAAGCAGAAGAACGGCATCAA

GGCAAACTTCAAGATCCGCCACAACATCGAGGACGGCGGCGTGCAGCTCGCCGACCACTACCAGCAGAACAC
CCCCATCGGCGACGGCCCCGTGCTGCTGCCCGACAACCACTACCTGAGCTACCAGTCCAAACTGAGCAAAGAC
CCCAACGAGAAGCGCGATCACATGGTCCTGCTGGAGTTCGTGACCGCCGCCGGGATCACTCTCGGCATGGAC
GAGCTGTACAAGGGATCCGGCGGAAGCGGCTCCATGTCCGACAGCGAGAAGCTCAACCTGGACTCGATCATC
GGGCGCCTGCTGGAAGTGCAGGGCTCGCGGCCTGGCAAGAATGTACAGCTGACAGAGAACGAGATCCGCGG
TCTGTGCCTGAAATCCCGGGAGATTTTTCTGAGCCAGCCCATTCTTCTGGAGCTGGAGGCACCCCTCAAGATCT
GCGGTGACATACACGGCCAGTACTACGACCTTCTGCGACTATTTGAGTATGGCGGTTTCCCTCCCGAGAGCAA
CTACCTCTTTCTGGGGGACTATGTGGACAGGGGCAAGCAGTCCTTGGAGACCATCTGCCTGCTGCTGGCCTAT
AAGATCAAGTACCCCGAGAACTTCTTCCTGCTCCGTGGGAACCACGAGTGTGCCAGCATCAACCGCATCTATG
GTTTCTACGATGAGTGCAAGAGACGCTACAACATCAAACTGTGGAAAACCTTCACTGACTGCTTCAACTGCCT
GCCCATCGCGGCCATAGTGGACGAAAAGATCTTCTGCTGCCACGGAGGCCTGTCCCCGGACCTGCAGTCTATG
GAGCAGATTCGGCGGATCATGCGGCCCACAGATGTGCCTGACCAGGGCCTGCTGTGTGACCTGCTGTGGTCT
GACCCTGACAAGGACGTGCAGGGCTGGGGCGAGAACGACCGTGGCGTCTCTTTTACCTTTGGAGCCGAGGTG
GTGGCCAAGTTCCTCCACAAGCACGACTTGGACCTCATCTGCCGAGCACACCAGGTGGTAGAAGACGGCTAC
GAGTTCTTTGCCAAGCGGCAGCTGGTGACACTTTTCTCAGCTCCCAACTACTGTGGCGAGTTTGACAATGCTG
GCGCCATGATGAGTGTGGACGAGACCCTCATGTGCTCTTTCCAGATCCTCAAGCCCGCCGACAAGAACAAGG
GGAAGTACGGGCAGTTCAGTGGCCTGAACCCTGGAGGCCGACCCATCACCCCACCCCGCAATTCCGCCAAAG
CCAAGAAATAGTAAAGCGGCCGCTAAG

DNA sequence encoding mVenus-Ctrl with HindIII and NotI restriction sites used for restriction digest and ligation into pcDNA/FRT/TO entry vector:

GTACAAGCTTGCCACCATGGTGAGCAAGGGCGAGGAGCTGTTCACCGGGGTGGTGCCCATCCTGGTCGAGCT
GGACGGCGACGTAAACGGCCACAAGTTCAGCGTGTCCGGCGAGGGCGAGGGCGATGCCACCTACGGCAAGC
TGACCCTGAAGTTCATCAGCACCACCGGCAAGCTGCCCGTGCCCTGGCCCACCCTCGTGACCACCCTCGGCTA
CGGCCTGCAGTGCTTCGCCCGCTACCCCGACCACATGAAGCAGCACGACTTCTTCAAGTCCGCCATGCCCGAA
GGCTACGTCCAGGAGCGCACCATCTTCTTCAAGGACGACGGCAACTACAAGACCCGCGCCGAGGTGAAGTTC
GAGGGCGACACCCTGGTGAACCGCATCGAGCTGAAGGGCATCGACTTCAAGGAGGACGGCAACATCCTGGG
GCACAAGCTGGAGTACAACTACAACAGCCACAACGTCTATATCACCGCCGACAAGCAGAAGAACGGCATCAA
GGCAAACTTCAAGATCCGCCACAACATCGAGGACGGCGGCGTGCAGCTCGCCGACCACTACCAGCAGAACAC
CCCCATCGGCGACGGCCCCGTGCTGCTGCCCGACAACCACTACCTGAGCTACCAGTCCAAACTGAGCAAAGAC
CCCAACGAGAAGCGCGATCACATGGTCCTGCTGGAGTTCGTGACCGCCGCCGGGATCACTCTCGGCATGGAC
GAGCTGTACAAGGGATCCGGCGGAAGCGGCTCCTAGTAAAGCGGCCGCTAAG

Amino acid sequence of mVenus-PP1α:

MVSKGEELFTGVVPILVELDGDVNGHKFSVSGEGEGDATYGKLTLKFISTTGKLPVPWPTLVTTLGYGLQCFARYPD
HMKQHDFFKSAMPEGYVQERTIFFKDDGNYKTRAEVKFEGDTLVNRIELKGIDFKEDGNILGHKLEYNYNSHNVYI
TADKQKNGIKANFKIRHNIEDGGVQLADHYQQNTPIGDGPVLLPDNHYLSYQSKLSKDPNEKRDHMVLLEFVTAA
GITLGMDELYKGSGGSGSMSDSEKLNLDSIIGRLLEVQGSRPGKNVQLTENEIRGLCLKSREIFLSQPILLELEAPLKIC
GDIHGQYYDLLRLFEYGGFPPESNYLFLGDYVDRGKQSLETICLLLAYKIKYPENFFLLRGNHECASINRIYGFYDECKR
RYNIKLWKTFTDCFNCLPIAAIVDEKIFCCHGGLSPDLQSMEQIRRIMRPTDVPDQGLLCDLLWSDPDKDVQGWG
ENDRGVSFTFGAEVVAKFLHKHDLDLICRAHQVVEDGYEFFAKRQLVTLFSAPNYCGEFDNAGAMMSVDETLMC
SFQILKPADKNKGKYGQFSGLNPGGRPITPPRNSAKAKK

Amino acid sequence of mVenus-Ctrl:

MVSKGEELFTGVVPILVELDGDVNGHKFSVSGEGEGDATYGKLTLKFISTTGKLPVPWPTLVTTLGYGLQCFARYPD
HMKQHDFFKSAMPEGYVQERTIFFKDDGNYKTRAEVKFEGDTLVNRIELKGIDFKEDGNILGHKLEYNYNSHNVYI
TADKQKNGIKANFKIRHNIEDGGVQLADHYQQNTPIGDGPVLLPDNHYLSYQSKLSKDPNEKRDHMVLLEFVTAA
GITLGMDELYKGSGGSGS

1.1.3. Cloning of pcDNA-FRT/TO-IRS2-mVenus

pcDNA-FRT/TO-IRS2-mVenus represents human IRS2 tagged with mVenus at its C terminus in a pcDNA-FRT/TO plasmid. Plasmid R777-E111 Hs.IRS2 was kindly gifted from Dominic Esposito (Addgene plasmid #70395) and was used as a template to amplify IRS2. IRS2 was first amplified with primers ATGGCTTCCCCCCCA (fw) and TTCCTTCACGATAGTTGCTTC (rev). The resulting PCR product was used as a template in PCR to introduce a Kozak sequence and AflII restriction site using primer CCTTCTTAAGACCACCATGGCTTCCCCCCCA (fw) and a GS linker and HindIII restriction site using primer CGTTAAGCTTGCGCCCACCTCCGCCGCTTCCACCGCCTCCTTCCTTCACGATAGTTGCTTC (rev). The resulting fragment was digested with AflII and HindIII and inserted into the AflII/HindIII-digested pcDNA-FRT/TO-mVenus entry vector. All PCRs described herein were run on a 0.8% agarose gel for analysis and all plasmids were verified in terms of sequence integrity using Sanger sequencing.

DNA sequence encoding IRS2 with AflII and HindIII restriction sites used for restriction digest and ligation into pcDNA/FRT/TO-mVenus entry vector:

CTTAAGACCACCATGGCTTCCCCCCCAAGGCACGGCCCCCCTGGACCTGCTTCTGGCGATGGACCTAATCTGA
ACAATAACAATAACAATAACAATCACTCCGTCCGGAAGTGCGGATACCTGAGAAAGCAGAAACATGGCCACA
AACGATTCTTTGTGCTGCGAGGACCAGGAGCAGGAGGGGACGAGGCTACCGCAGGAGGAGGAAGCGCTCCT
CAGCCACCTAGACTGGAATACTATGAAAGTGAGAAGAAATGGAGGTCAAAGGCAGGAGCTCCAAAACGCGT
GATCGCACTGGACTGCTGTCTGAACATTAATAAGAGGGCAGATGCCAAGCACAAATACCTGATCGCCCTGTAT
ACTAAAGATGAGTACTTTGCTGTCGCCGCTGAAAACGAGCAGGAACAGGAGGGCTGGTATCGAGCACTGACC
GACCTGGTGTCCGAAGGACGAGCAGCAGCTGGAGATGCTCCACCAGCAGCAGCTCCAGCAGCATCATGCAGC
GCATCCCTGCCAGGAGCTCTGGGAGGATCTGCAGGAGCTGCAGGAGCAGAGGACAGTTATGGACTGGTGGC
TCCAGCAACAGCCGCTTACCGCGAAGTGTGGCAGGTCAACCTGAAGCCCAAAGGGCTGGGACAGTCTAAGAA
TCTGACCGGAGTGTACAGGCTGTGCCTGAGCGCACGCACAATCGGATTCGTGAAACTGAACTGTGAGCAGCC
CAGCGTCACCCTGCAGCTGATGAATATCCGGAGATGTGGCCACTCTGATAGTTTCTTTTTCATTGAAGTGGGA
AGATCCGCAGTCACAGGACCTGGGGAACTGTGGATGCAGGCTGACGATTCTGTGGTCGCACAGAACATCCAT
GAAACTATTCTGGAGGCCATGAAGGCTCTGAAAGAACTGTTTGAGTTCCGCCCCCGATCAAAGAGCCAGAGC
TCCGGATCTAGTGCAACCCACCCTATTAGCGTGCCAGGAGCAAGGCGACACCATCACCTGGTCAATCTGCCTC
CATCCCAGACAGGCCTGGTGCGACGGTCACGAACTGACAGCCTGGCAGCAACCCCACCTGCTGCAAAATGCT
CAAGCTGTCGGGTGAGAACAGCCTCCGAGGGCGATGGGGGAGCAGCTGCAGGAGCAGCTGCAGCAGGAGC
ACGGCCTGTGTCTGTCGCTGGGAGTCCACTGTCACCAGGACCCGTGAGAGCCCCTCTGTCCAGGTCTCATACT
CTGAGCGGAGGATGCGGAGGAAGAGGATCCAAGGTCGCACTGCTGCCAGCAGGAGGAGCACTGCAGCATA
GTAGGTCAATGAGCATGCCAGTGGCACACAGCCCACCAGCTGCAACCAGCCCAGGATCCCTGTCCTCTAGTTC
AGGACACGGCTCCGGATCTTATCCTCCACCACCTGGACCACATCCACCACTGCCTCACCCACTGCATCACGGAC
CAGGACAGAGGCCTAGCTCCGGAAGTGCATCAGCTAGCGGATCCCCATCTGATCCCGGCTTCATGTCCCTGGA
CGAGTACGGCTCTAGTCCTGGGGACCTGCGAGCCTTCTGCTCTCATCGGAGTAACACCCCAGAAAGCATCGCT
GAGACACCTCCAGCAAGAGACGGAGGAGGAGGAGGAGAATTTTACGGCTATATGACAATGGATCGGCCACT
GAGTCACTGTGGGAGATCATATAGAAGGGTGAGCGGAGACGCAGCTCAGGACCTGGATAGGGGCCTGAGG
AAGCGCACTTACTCTCTGACCACACCAGCACGACAGCGACCAGTGCCTCAGCCATCAAGCGCTAGCCTGGATG
AGTATACCCTGATGCGAGCAACATTCAGTGGCTCAGCCGGGAGGCTGTGCCCTTCCTGTCCAGCTTCCTCTCCC
AAAGTGGCATACCATCCCTATCCTGAAGACTATGGAGATATCGAGATTGGCAGCCACCGGAGTTCAAGCTCCA
ATCTGGGAGCCGACGATGGCTACATGCCAATGACCCCTGGAGCAGCACTGGCTGGAAGCGGATCCGGCTCTT
GTCGCAGTGACGATTACATGCCAATGTCACCCGCCAGTGTGTCAGCTCCCAAGCAGATCCTGCAGCCTCGAGC
TGCAGCAGCTGCAGCAGCTGCAGTGCCATCCGCTGGACCAGCAGGACCAGCACCCACATCAGCCGCTGGCCG
CACTTTTCCTGCCAGTGGAGGCGGGTATAAAGCATCTAGTCCTGCCGAATCAAGCCCAGAGGACAGCGGCTA
CATGCGGATGTGGTGCGGGTCAAAGCTGAGCATGGAGCATGCCGATGGCAAACTGCTGCCTAACGGGGACT
ACCTGAATGTGAGCCCATCCGATGCAGTCACTACCGGAACCCCACCTGACTTCTTCAGCGCAGCACTGCACCCT
GGAGGAGAACCACTGCGAGGAGTGCCCGGATGCTGTTATTCCTCTCTGCCACGGAGCTACAAGGCCCCCTAT
ACATGTGGGGGAGACTCCGATCAGTACGTGCTGATGAGTTCACCCGTCGGAAGAATTCTGGAGGAAGAGAG
GCTGGAGCCTCAGGCAACTCCTGGACCATCCCAGGCTGCATCTGCTTTTGGGGCAGGACCTACCCAGCCACCA

CATCCAGTGGTCCCATCCCCTGTCCGCCCATCTGGAGGACGACCTGAGGGCTTCCTGGGACAGAGAGGAAGG
GCCGTGAGGCCTACACGACTGAGCCTGGAGGGCCTGCCTTCCCTGCCATCTATGCACGAATATCCACTGCCTC
CAGAGCCCAAGTCCCCTGGCGAATACATCAACATTGACTTTGGCGAGCCAGGAGCAAGACTGTCCCCACCTGC
ACCACCACTGCTGGCTTCTGCAGCTAGCTCCTCTAGTCTGCTGAGCGCTTCAAGCCCAGCATCCTCTCTGGGAT
CCGGAACACCTGGCACTAGTTCAGACTCTCGCCAGCGAAGTCCACTGTCAGATTATATGAATCTGGACTTCAG
CTCCCCAAAGTCCCCAAAACCAGGAGCACCAAGCGGACACCCTGTGGGATCCCTGGATGGACTGCTGTCTCCA
GAGGCCTCTAGTCCCTATCCTCCACTGCCCCCTAGACCATCTGCTAGTCCCTCAAGCTCCCTGCAGCCACCTCCT
CCACCACCTGCACCAGGAGAACTGTACAGGCTGCCACCAGCAAGCGCAGTGGCTACCGCACAGGGACCTGGA
GCAGCATCTAGTCTGTCAAGCGACACTGGAGATAACGGCGACTACACCGAGATGGCCTTTGGCGTGGCTGCA
ACTCCTCCACAGCCCATCGCCGCTCCCCCTAAGCCTGAAGCAGCAAGAGTGGCAAGCCCTACCTCCGGAGTCA
AAAGGCTGAGTCTGATGGAGCAGGTGTCAGGGGTCGAAGCCTTCCTGCAGGCTTCCCAGCCACCAGACCCTC
ATCGAGGCGCAAAAGTGATCCGAGCAGATCCACAGGGAGGACGACGACACTCCTCTGAGACCTTCAGCT
CAACAACTACCGTGACTCCAGTCTCACCCAGCTTCGCTCATAACCCCAAGAGACACAATTCCGCATCTGTGGAG
AATGTCTCTCTGAGGAAAAGCTCCGAAGGAGGAGTGGGAGTCGGACCAGGAGGAGGAGACGAGCCTCCAAC
CAGCCCTCGACAGCTGCAGCCAGCACCCCCTCTGGCCCCCCAGGGACGGCCTTGGACACCAGGACAGCCAGG
AGGACTGGTGGGATGCCCAGGATCTGGAGGAAGTCCTATGAGAAGGGAGACCAGCGCCGGATTCCAGAACG
GCCTGAATTACATCGCTATTGACGTGCGGGAAGAGCCAGGACTGCCACCACAGCCACAGCCACCACCACCTCC
ACTGCCTCAGCCAGGGGATAAGTCTAGTTGGGGACGGACAAGAAGCCTGGGAGGACTGATCTCCGCAGTGG
GAGTCGGATCTACTGGAGGAGGATGTGGAGGACCAGGACCTGGAGCTCTGCCCCCTGCAAATACCTACGCAT
CCATTGACTTTCTGTCCCATCATCTGAAGGAAGCAACTATCGTGAAGGAAGGAGGCGGTGGAAGCGGCGGAG
GTGGGCGCAAGCTT

Amino acid sequence of IRS2-mVenus:
MASPPRHGPPGPASGDGPNLNNNNNNNNHSVRKCGYLRKQKHGHKRFFVLRGPGAGGDEATAGGGSAPQPP
RLEYYESEKKWRSKAGAPKRVIALDCCLNINKRADAKHKYLIALYTKDEYFAVAAENEQEQEGWYRALTDLVSEGRA
AAGDAPPAAAPAASCSASLPGALGGSAGAAGAEDSYGLVAPATAAYREVWQVNLKPKGLGQSKNLTGVYRLCLS
ARTIGFVKLNCEQPSVTLQLMNIRRCGHSDSFFFIEVGRSAVTGPGELWMQADDSVVAQNIHETILEAMKALKELF
EFRPRSKSQSSGSSATHPISVPGARRHHHLVNLPPSQTGLVRRSRTDSLAATPPAAKCSSCRVRTASEGDGGAAAG
AAAAGARPVSVAGSPLSPGPVRAPLSRSHTLSGGCGGRGSKVALLPAGGALQHSRSMSMPVAHSPPAATSPGSLS
SSSGHGSGSYPPPPGPHPPLPHPLHHGPGQRPSSGSASASGSPSDPGFMSLDEYGSSPGDLRAFCSHRSNTPESIAE
TPPARDGGGGGEFYGYMTMDRPLSHCGRSYRRVSGDAAQDLDRGLRKRTYSLTTPARQRPVPQPSSASLDEYTL
MRATFSGSAGRLCPSCPASSPKVAYHPYPEDYGDIEIGSHRSSSSNLGADDGYMPMTPGAALAGSGSGSCRSDDY
MPMSPASVSAPKQILQPRAAAAAAAAVPSAGPAGPAPTSAAGRTFPASGGGYKASSPAESSPEDSGYMRMWCG
SKLSMEHADGKLLPNGDYLNVSPSDAVTTGTPPDFFSAALHPGGEPLRGVPGCCYSSLPRSYKAPYTCGGDSDQYV
LMSSPVGRILEEERLEPQATPGPSQAASAFGAGPTQPPHPVVPSPVRPSGGRPEGFLGQRGRAVRPTRLSLEGLPSL
PSMHEYPLPPEPKSPGEYINIDFGEPGARLSPPAPPLLASAASSSSLLSASSPASSLGSGTPGTSSDSRQRSPLSDYMN
LDFSSPKSPKPGAPSGHPVGSLDGLLSPEASSPYPPLPPRPSASPSSSLQPPPPPPAPGELYRLPPASAVATAQGPGA
ASSLSSDTGDNGDYTEMAFGVAATPPQPIAAPPKPEAARVASPTSGVKRLSLMEQVSGVEAFLQASQPPDPHRGA
KVIRADPQGGRRRHSSETFSSTTTVTPVSPSFAHNPKRHNSASVENVSLRKSSEGGVGVGPGGGDEPPTSPRQLQP
APPLAPQGRPWTPGQPGGLVGCPGSGGSPMRRETSAGFQNGLNYIAIDVREEPGLPPQPQPPPPPLPQPGDKSS
WGRTRSLGGLISAVGVGSTGGGCGGPGPGALPPANTYASIDFLSHHLKEATIVKEGGGGSGGGGRKLASMVSKGE
ELFTGVVPILVELDGDVNGHKFSVSGEGEGDATYGKLTLKFISTTGKLPVPWPTLVTTLGYGLQCFARYPDHMKQH
DFFKSAMPEGYVQERTIFFKDDGNYKTRAEVKFEGDTLVNRIELKGIDFKEDGNILGHKLEYNYNSHNVYITADKQK
NGIKANFKIRHNIEDGGVQLADHYQQNTPIGDGPVLLPDNHYLSYQSKLSKDPNEKRDHMVLLEFVTAAGITLGM
DELYK

1.2. Cell Culture, transfection and generation of stable cell lines

Cell culture and transfection was carried out as described previously.[1] Stable cell lines were generated by cultivating parental HeLa Kyoto FlpInTRex cells (provided by the Hentze Lab at EMBL Heidelberg) in general growth medium (DMEM, 1 g/L glucose, GlutaMAX™ Supplement, 1 mM pyruvate, Gibco) and keeping them under selection with 5 µg/mL Blasticidine and 200 µg/mL Zeocine. Two 10 cm cell

culture dishes at 60% confluency in general medium without antibiotics were then transfected with 0.5 µg pcDNA5/FRT/TO-mVenus-PPP1CA or pcDNA5/FRT/TO-mVenus together with 4 µg pOG44 and 27 µL FuGene HD (Promega) in 600 µL OptiMEM (Gibco). 24 h post transfection, selection was started with 5 µg/mL blasticidine and 0.2 mg/mL hygromycine B. After successful formation of colonies, cells were harvested and seeded on new dishes. Next, cells were induced with 1 µg/mL doxycycline for 24 h and subjected to cell sorting at the CCI Lighthouse Facility of the University Clinics Freiburg. The resulting cell stock was used for further experiments.

1.3. Cell Culture Experiments

1.3.1. Co-immunoprecipitation experiment with mVenus-PDP1(m)

Cell growth and Co-immunoprecipitation experiment with mVenus-PDP1(m). Eight 10 cm cell culture dishes with HeLa Kyoto cells (BIOSS cell line repository) were grown to 60% confluency and transfected with 10 µg pTriEx-mVenus-PDP1 or pTriEx-mVenus-PDP1m per plate using FuGene HD (4 plates per plasmid). 24 h post transfection cells were placed on ice and washed twice with 10 mL ice-cold PBS. After the final wash, dishes were placed at a 45° angle and after 30 s, excess PBS was again removed. Cells were then lysed in 500 µL lysis buffer per plate (100 mM NaCl, 10 mM Tris-HCl pH 7.5, 0.1% IGEPAL, 1 mM EDTA, 1 mM EGTA, 1x PhosStop (Roche), 1x Protease Inhibitor) and cells were scratched from the plate using a cell scraper (Sarstedt) and pressed through a needle (21 G, BD) 8 times, before being transferred to an Eppendorf tube. Next, all tubes were centrifuged at 4 °C and 10^4 rcf for 10 min. 20 µL of the resulting supernatant served as input sample. 25 µL CnBr-GFP nanobody agarose bead slurry (EMBL PEPcore) per tube (8 tubes total) were washed two times with 1 mL lysis buffer (centrifugation at 4 °C and 300 rcf for 1 min). Beads were then incubated with the resulting supernatant from lysate centrifugation on a rotation wheel at 4 °C for 2 h. Beads were then washed three times with 1 mL lysis buffer (centrifugation at 4 °C and 300 rcf for 1 min) and eluted in 40 µL 1x LDS sample buffer (NuPAGE; Thermo Fisher) with 100 mM DTT by heating to 70 °C for 10 min. Samples were then immediately stored at -80 °C for subsequent MS analysis. One repeat per plasmid was loaded onto a 10-well NuPAGE™ 4 to 12% Bis-Tris 1.0 mm mini protein gel (Invitrogen), run, and stained with colloidal Coomassie for analysis.

Mass spectrometric sample preparation for co-immunoprecipitation. In-gel trypsin digestion of all co-immunoprecipitated samples was performed according to standard procedures.[2] Briefly, the samples were run on a Nu-PAGE™ 4%–12% Bis-Tris protein gel (Thermo Fisher Scientific) for about 1 cm. Subsequently, the still not size-separated single protein band per sample was cut, reduced (50 mM dithiothreitol), alkylated (55 mM chloroacetamide) and digested overnight with trypsin (Trypsin Gold, mass spectrometry grade, Promega). The peptides obtained were dried to completeness and resuspended in 25 µL of buffer A (2% ACN, 0.1% formic acid in HPLC grade water), and 5 µL of sample were injected per MS measurement.

Mass spectrometric data acquisition for co-immunoprecipitation. LC-MS/MS measurements were performed on a Dionex Ultimate 3000 RSLCnano system coupled to a Q-Exactive HF-X mass spectrometer (Thermofisher Scientific, Bremen). Injected peptides were delivered to a trap column (ReproSil-pur C18-AQ, 5 µm, Dr. Maisch, 20 mm × 75 µm, self-packed) at a flow rate of 5 µL/min in 100% solvent A (0.1% formic acid in HPLC grade water). After 10 minutes of loading, peptides were transferred to an analytical column (ReproSil Gold C18-AQ, 3 µm, Dr. Maisch, 450 mm × 75 µm, self-packed) and separated using a 50 min gradient from 4% to 32% of solvent B (0.1% FA, 5% DMSO in ACN) in solvent A (0.1% FA, 5% DMSO in HPLC grade water) at 300 nL/min flow rate. The Q-Exactive HF-X mass spectrometer was operated in data dependent acquisition (DDA) and positive ionization mode. MS1 spectra (360–1300 m/z) were recorded at a resolution of 60k using an automatic gain

control (AGC) target value of 3e6 and maximum injection time (maxIT) of 45 ms. Up to 18 peptide precursors were selected for fragmentation. Only precursors with charge state 2 to 6 were selected and dynamic exclusion of 25 s was enabled. Peptide fragmentation was performed using higher energy collision induced dissociation (HCD) and a normalized collision energy (NCE) of 26%. The precursor isolation window width was set to 1.3 m/z. MS2 Resolution was 15.000 with an automatic gain control (AGC) target value of 1e5 and maximum injection time (maxIT) of 25 ms (full proteome).

Mass spectrometric data analysis for co-immunoprecipitation. Peptide identification and quantification was performed using the software MaxQuant[3] (version 1.6.1.0) with its built-in search engine Andromeda.[4] MS2 spectra were searched against all canonical human protein sequences as annotated in the Swissprot reference database (20205 protein entries, downloaded 22nd of March 2016, internally annotated with PFAM domains), supplemented with the amino acid sequence of the bait protein (mVenus-PDP1(m)) as well as with common contaminants (built-in option in MaxQuant). Trypsin/P was specified as proteolytic enzyme. Carbamidomethylated cysteine was set as fixed modification. Oxidation of methionine, acetylation at the protein N-terminus and phosphorylation on S, T, and Y were specified as variable modifications. Results were adjusted to 1% false discovery rate on peptide spectrum match level and protein level employing a target-decoy approach using reversed protein sequences. The MaxQuant output (proteinGroups.txt) was normalized based on the total bait protein intensity per sample. The resulting dataset was imported into the software suite Perseus[5] (version 1.5.8.5) and proteins only identified by site, reverse proteins and potential contaminants were removed, reducing the originally imported 2,111 proteins to 1,961. Next, all protein intensities were log2 transformed and further filtered for at least two unique detected peptides over all three replicates of at least one condition. This reduced the dataset to 1,056 detectable proteins. Subsequently, N/A values were imputed from a normal distribution (width 0.3, down shift 1.8). The resulting dataset was the basis for subsequent quantitative comparison by a Student's t-test adjusted for multiple-hypothesis testing (250 randomizations, FDR 1%, s0: 0.5) to find proteins differentially binding to mVenus-PDP1 and mVenus-PDP1m, respectively. Results were exported and final plots were produced in R using ggplot2 and the tidyverse package. For total proteome intensities, protein intensities of each individual protein prior to imputation were summed across all replicates of one condition, provided the protein was found in all three replicates. The same was done for the dataset obtained from the co-immunoprecipitation experiment using mVenus-Ctrl described below. Both datasets were combined and proteins also pulled down with mVenus-Ctrl were excluded as potential non-specific binders. Data from the CRAPome database[6] obtained by filtering for AP-MS single step epitope tag AP-MS experiments in HeLa cells (18 experiments, accessed 17th Jan 2023) was also used to exclude proteins that are common contaminants. The resulting list was used to identify proteins that are potential non-specific binders of PDPs. Final plots were produced in R using ggplot2 and the tidyverse package.

1.3.2. Co-immunoprecipitation experiment with mVenus-PP1α after PDP(m)-*Nal* treatment

Culturing and co-immunoprecipitation with mVenus-PP1α after PDP(m)-Nal treatment for mass spectrometry. Eight 10 cm dishes for the cell line HeLa Kyoto FlpInTrex mVenus-PP1α and four 10 cm dishes of HeLa Kyoto FlpInTrex mVenus-Ctrl were induced with 1 µg/mL doxycycline for 24 h. Lyophilized stocks of PDP-*Nal* and PDPm-*Nal* (jpt, Heidelberg) were dissolved in 10% DMSO to a stock concentration of 10 mM and stored at –20 °C. On the day of the experiment, peptides were diluted to final concentration of 50 µM in growth medium (DMEM, 1 g/L glucose, GlutaMAX™ Supplement, 1 mM pyruvate, Gibco). Five minutes before lysis, medium was removed from the cell culture dishes and 50 µM PDP-*Nal* or PDPm-*Nal* in 5 mL growth medium was added onto the dishes for HeLa Kyoto

FlpInTrex mVenus-PPP1CA (4 plates per peptide variant). For incubation, cells were placed back at 37 °C. After 5 min, cells were immediately placed on ice and washed three times with 10 mL ice-cold PBS. After final removal of PBS, plates were placed at a 45° angle for 1 min to collect and remove excess PBS. Cell lysis was then carried out by adding 500 µL lysis buffer per dish (100 mM NaCl, 10 mM Tris-HCl pH 7.5, 1 mM EDTA, 1 mM EGTA, 0.1% IGEPAL, 1x cOmplete Mini protease inhibitor cocktail (Roche)). Cells were then scraped on ice, transferred to an Eppendorf tube and additional lysis was carried out by pushing cells through an injection needle (21 G, BD) eight times. Subsequently, cells were centrifuged at 4 °C and 10^4 rcf for 10 min. From the resulting supernatant, 50 µL were removed as input sample. The remainder was used for immunoprecipitation. The lysate from each plate was incubated with 25 µL agarose GFP-Trap beads (Chromotek, prewashed in lysis buffer) on a rotation wheel at 4 °C for 2h. After three washes (spin down at 4 °C and 400 rcf for 1 min) with 1 mL lysis buffer, samples were eluted by incubation of bead slurry with 30 µL 1x LDS sample buffer (NuPAGE; Thermo Fisher) at 70 °C for 10 min. One replicate was loaded onto a 10-well NuPAGE™ 4 to 12% Bis-Tris 1.0 mm mini protein gel (Invitrogen)and analyzed by Colloidal Coomassie,[7] the remaining 3 replicates were frozen at –80 °C for analysis by mass spectrometry.

Mass spectrometric sample preparation for co-immunoprecipitation. In-gel trypsin digestion of all co-immunoprecipitated samples was performed as described above for mVenus-PDP1(m) samples.

Mass spectrometric data acquisition for co-immunoprecipitation. LC-MS/MS measurements were performed on a Dionex Ultimate 3000 RSLCnano system coupled to an Orbitrap Fusion Lumos Tribrid mass spectrometer (Thermofisher Scientific, Bremen). Injected peptides were delivered to a trap column (ReproSil-pur C18-AQ, 5 µm, Dr. Maisch, 20 mm × 75 µm, self-packed) at a flow rate of 5 µL/min in 100% solvent A (0.1% formic acid in HPLC grade water). After 10 minutes of loading, peptides were transferred to an analytical column (ReproSil Gold C18-AQ, 3 µm, Dr. Maisch, 400 mm × 75 µm, self-packed) and separated using a 50 min gradient from 4% to 32% of solvent B (0.1% FA, 5% DMSO in ACN) in solvent A (0.1% FA, 5% DMSO in HPLC grade water) at 300 nL/min flow rate. The mass spectrometer was operated in data dependent acquisition and positive ionization mode. MS1 spectra (360–1300 m/z) were recorded at a resolution of 60k using an automatic gain control (AGC) target value of 4e5 and maximum injection time (maxIT) of 50 ms. MS2 spectra of up to 20 precursor peptides were acquired at a resolution of 15k with an automatic gain control (AGC) target value of 5e4 and maximum injection time (maxIT) of 22 ms. The precursor isolation window width was set to 1.3 m/z and fragmentation was performed using higher energy collision induced dissociation (HCD) and a normalized collision energy of 30%. Dynamic exclusion was enabled with 20 s exclusion time (mass tolerance +/-10 ppm). Precursors that were singly-charged, unassigned or with charge states >6+ were excluded for fragmentation.

Mass spectrometric data analysis for co-immunoprecipitation. Peptide identification and quantification was performed using the software MaxQuant[3] (version 1.6.3.4) with its built-in search engine Andromeda.[4] MS2 spectra were searched against all canonical human protein sequences as annotated in the Swissprot reference database (20205 protein entries, downloaded 22nd of March 2016, internally annotated with PFAM domains), supplemented with the amino acid sequence of the bait protein (mVenus_PPP1CA) as well as with common contaminants (built-in option in MaxQuant). Trypsin/P was specified as proteolytic enzyme. Carbamidomethylated cysteine was set as fixed modification. Oxidation of methionine and acetylation at the protein N-terminus was specified as variable modifications. Results were adjusted to 1% false discovery rate on peptide spectrum match level and protein level employing a target-decoy approach using reversed protein sequences. The proteingroup.txt MaxQuant output was imported into the software suite Perseus[5] (version 1.5.8.5) and column types assigned, resulting in a dataset of 2,224 proteins. Next, proteins only identified by

site, reverse proteins and potential known contaminants were filtered out. LFQ intensity values were then transformed into log2 and treatment conditions were assigned as categorical annotations Ctrl, PDPm-*Nal,* and PDP-*Nal* to the nine samples (three biological replicates per condition). The dataset was further reduced by filtering for proteins without any missing value in at least one of the three treatments. N/A values were imputed from a normal distribution (width 0.3, down shift 1.8). This final dataset of 1,311 proteins entered final quantitative analysis. To this end, LFQ intensities of conditions PDPm-*Nal* vs. Ctrl and PDP-*Nal* vs. PDPm-*Nal* were compared by a two-sided unpaired Student's t-test adjusted for multiple-hypothesis testing (250 randomizations, FDR 5%, s0: 0.1). The results were exported and final plots were produced in R using ggplot2 and the tidyverse package. All identified proteins were compared to the set of proteins annotated as PPP1R in the HGNC database,[8] interactors listed in DEPOD[9] and proteins reported to bind PP1 through an RVxF motif in the KVxF PP1 docking motif repository.[10]

Culturing and co-immunoprecipitation with mVenus-PPP1CA after PDP(m)-Nal treatment for immunoblotting. For each of the six experiments, three 10 cm dishes for the cell line HeLa Kyoto FlpInTrex mVenus-PP1α and one 10 cm dish of HeLa Kyoto FlpInTrex mVenus-Ctrl were induced with 1 µg/mL Doxycycline for 24 h. Peptide treatment and lysis was carried out as for the mass spectrometry experiment. From the clarified lysate, 50 µL were removed as input sample, combined with 2x reducing SDS sample buffer (50 µL) and boiled at 95 °C for 10 min. The remaining lysate was used for immunoprecipitation and incubated with 25 µL magnetic agarose GFP-Trap beads (Chromotek, prewashed with 3 x 500 µL lysis buffer) per plate at 4 °C for 2 h on a rotation wheel. The supernatant was removed and after three washes with 1 mL lysis buffer, samples were eluted by incubation of the beads with 30 µL 2x reducing SDS sample buffer (125 mM Tris-HCl pH 6.8, 20% v/v glycerol, 4% w/v SDS, 0.02% w/v bromophenol blue, 5% v/v β-mercaptoethanol, 1 mM DTT) at 95 °C for 10 min.

Immunoblotting and quantification. Input samples (25 µL) and eluate samples (30 µL) were separated on 10-well NuPAGE™ 4 to 12% Bis-Tris 1.0 mm mini protein gels (Invitrogen) in MOPS buffer at 180 V. Proteins were transferred onto Immobilon-FL PVDF blotting membranes (Merck Millipore) by wet blotting at 100 V for 1 h. Membranes were blocked with Tris-buffered saline with 0.1% Tween 20 (TBS-T) containing 5% (w/v) non-fat milk for 45 min at room temperature. After three washes with TBS-T (10 min), membranes were then incubated with α-RMP (URI1) antibody (1:1000, CST, #5844) in TBS-T with 5% BSA overnight at 4 °C. Afterwards blots were washed three times in TBS-T (15 min) and incubated with donkey anti-rabbit IgG-horseradish peroxidase (HRP) conjugate (1:5000, Merck Millipore, GENA934) in TBS-T with 5% (w/v) non-fat milk for 1 h at room temperature. After three final washes with TBS-T (10 min), blots were developed using Western Lightning Plus-ECL Enhanced Chemiluminescence Substrate (Perkin Elmer) and a Fusion FX Imaging System (Vilber). Following imaging, the membrane was stripped using mild stripping buffer[11] (15 g/L glycine, 1 g/L SDS, 1% v/v Tween 20, pH 2.2 at 80% of final volume) by incubating twice with mild stripping buffer (10 min), washing twice with PBS (10 min) and washing twice with TBS-T (5 min). The membrane was then blocked as above, washed as above and incubated with α-GFP antibody (1:2500, abcam, ab6556) in TBS-T with 5% BSA overnight at 4 °C. The membrane was washed, incubated with secondary antibody and imaged as described above. All blots were quantified using raw images and FIJI software (version 1.53q, National Institutes of Health, USA). Files were analyzed without further modifications using the gel analysis tool. After plotting the lanes, the band profiles were inspected and the baseline was adjusted where necessary. The area measurements were exported from the analysis table and used to calculate the ratio of the URI signal to the GFP signal. This ratio was used for statistical analysis in GraphPad Prism (version 6.05, GraphPad Software). All ratios obtained from the same membrane, i.e.

from the same replicate, were treated as matched data and a Tukey multiple comparisons test was carried out. The p-values shown are multiplicity-adjusted.

1.3.3. PDP(m)-*Nal* treatment and sample preparation for phosphoproteomics

Culturing and cell lysis for PDP(m)-*Nal* phosphoproteome analysis. Lyophilized stocks of PDP-*Nal* and PDPm-*Nal* (jpt, Heidelberg) were dissolved in 10% DMSO to a stock concentration of 10 mM and stored at –20 °C. On the day of the experiment, peptides were diluted to final concentration of 50 µM in growth medium (DMEM, 1 g/L glucose, GlutaMAX™ Supplement, 1 mM pyruvate, Gibco). As control, a 10% DMSO solution without peptides was diluted in growth medium in the same manner. HeLa Kyoto cells (obtained from the BIOSS cell line repository) were grown to 90% confluency in 10 cm cell culture dishes before being washed twice with 10 mL PBS. Dishes were placed at a 45° angle and excess PBS was again removed in order to avoid dilution during the following incubation step. Next, three dishes per condition were treated with 3 mL of 50 µM PDP-*Nal*, PDPm-*Nal,* or control solution described above. Subsequently, all dishes were placed back at 37 °C in the cell culture incubator for 5 min. Dishes were then immediately placed on ice, washed two times with ice-cold PBS and lysed in 500 µL 8 M urea lysis buffer (8 M urea, 40 mM Tris-HCl pH 7.6, 20 nM Calyculin A, 1x PhosStop (Roche) and cOmplete EDTA-free protease inhibitor (#04693132001, Roche)) per dish. The lysate was collected by using a cell scraper and pressed through an injection needle (21 G, BD) using a syringe 8 times. 5 µL of sample were used for Bradford determination of total protein and the remaining sample was immediately frozen at -80°C for later MS analysis.

Mass spectrometric sample preparation for phosphoproteomics. 200 µg of protein lysates per sample were reduced (10 mM DTT, 30 min at 30 °C), alkylated (55 mM chloroacetamide, room temperature, 30 min, in the dark) and diluted to 1.3 M Urea with 50 mM NH_4HCO_3. Digestion was performed by adding trypsin (Promega, Trypsin Gold mass spectrometry grade, 1:50 enzyme-to-substrate ratio) and incubating overnight at 37 °C. Digests were acidified with 1% formic acid (FA) and desalted using 50 mg tC18 reversed-phase (RP) solid-phase extraction cartridges (Sep-Pak, Waters, WAT054960). Peptide solutions were dried in a SpeedVac (Labconco) and frozen at −80 °C until further usage. TMT labeling was carried out by reconstituting peptide solutions (200 µg peptide amount per sample) in 20 µL of 100 mM HEPES buffer (pH 8.5), and 5 µL of a specific 11.6 mM TMT reagent (Thermo Fisher Scientific) in 100% anhydrous ACN. After incubation at 25 °C and 400 rpm for 1 h, the labeling reaction was stopped by adding 2 µL of 5% hydroxylamine and incubating at 25 °C and 400 rpm for 15 min.[12] In total nine different peptide samples (biological triplicates of “control”, “PDP-*Nal*” and “PDPm-*Nal*”), each labeled with a specific TMT reagent, were pooled into a TMT-9-plex pool and acidified using 20 µL of 10% FA. Reaction vessels, in which the labeling took place were rinsed with 20 µL of 10% FA in 10% ACN (25 °C and 400 rpm, 5 min,), and the solvent was added to the pooled sample. The pools were dried down in a SpeedVac (Labconco) and desalted using 500 mg tC18, RP solid-phase extraction cartridges (Sep-Pak, Waters, WAT020805). Peptide solutions were dried in a SpeedVac and frozen at −80 °C. Phosphopeptides were enriched from 1.8 mg of TMT-labeled peptide pool using Fe-IMAC as described previously.[13] The Fe-IMAC eluate was desalted using C18 StageTips, and dried down in a SpeedVac (Labconco). High-pH RP tip fractionation into 6 fractions was performed in Stage Tips as previously described.[14] Tips were washed using 250 µL of 100% ACN, followed by 250 µL of 50% ACN in 25 mM NH_4HCO_2, pH 10 and equilibrated with 250 µL of 25mM NH_4HCO_2, pH 10. Subsequently, the phosphopeptides were reconstituted in 50 µL of 25mM NH_4HCO_2, pH 10, and loaded onto the C18

material. Peptides were eluted using 40 µL of solvent with increasing concentrations of ACN (5, 7.5, 10, 12.5, 15, 17.5 and 50% ACN) in 25mM NH_4HCO_2, pH 10. The 5 and 50% ACN fractions were pooled and the 17.5% ACN fraction was combined with the previously stored flow-through, resulting in a total of six fractions, which were dried and stored at −20 °C until LC-MS/MS measurement.

Mass spectrometric data acquisition for phosphoproteomics. LC-MS/MS measurements of TMT-labeled phosphopeptides were carried out using a Dionex Ultimate 3000 RSLCnano system coupled to an Orbitrap Fusion Lumos Tribrid mass spectrometer (Thermofisher Scientific, Bremen). Peptides were dissolved in 15 µL citrate solution (0.5% FA and 50 mM citric acid) and one third was injected. Peptides were delivered to a trap column (ReproSil-pur C18-AQ, 5 µm, Dr. Maisch, 20 mm × 75 µm, self-packed). After 10 minutes of loading, peptides were transferred to an analytical column (ReproSil Gold C18-AQ, 3 µm, Dr. Maisch, 400 mm × 75 µm, self-packed) applying a flow rate of 300 nL/min. Peptides were separated using a 90 min linear gradient from 4% to 32% solvent B (0.1% FA, 5% DMSO in ACN) in solvent A (0.1% FA, 5% DMSO in HPLC grade water). The Fusion Lumos was operated in data dependent acquisition (DDA), positive ionization and multi-notch MS3 mode.[15] Full scan MS1 spectra were recorded over a range of 360-1500 m/z at a resolution of 60k using an automatic gain control (AGC) target value of 4e5 and maximum injection time (maxIT) of 50 ms. Precursor ions were filtered according to charge state (2-6), dynamic exclusion (90 s with a ±10 ppm window), and monoisotopic precursor selection. MS2 spectra for peptide identification were recorded in the Orbitrap (Quadrupole isolation window 0.7 m/z). Peptide fragmentation was performed using higher energy collision induced dissociation (HCD) and a normalized collision energy (NCE) of 30% (AGC target value 5e4, maximum injection time 54 ms, resolution 30k). For TMT reporter ion quantification an additional MS3 spectrum was acquired in the Orbitrap over an m/z range of 100-1000 m/z at 50k resolution (AGC target value 1.2e5, maximum injection time 120 ms). For this, fragment ions were selected by multi-notch isolation, allowing a maximum of 10 notches. MS1 precursor isolation was performed with an MS1 isolation window of 1.2 m/z and an MS2 isolation window of 2 m/z. Fragmentation in the Orbitrap was performed by HCD at 55% NCE. The overall cycle time of the method was 3 s.

Data analysis for phosphoproteomics after PDP(m)-*Nal* treatment. Phosphopeptides were identified and quantified using MaxQuant[3] (v1.6.0.16) with enabled MS3-based TMT10plex quantification and default parameters. The data was searched against all canonical human protein sequences as annotated in the Swissprot reference database (20205 protein entries, downloaded 22nd of March 2016, internally annotated with PFAM domains) using the embedded search engine Andromeda[4]. Oxidation of methionine and phosphorylation on Ser, Thr, and Tyr were specified as variable modifications. Carbamidomethylation on cysteines was specified as a fixed modification. Trypsin (Trypsin/P) specified as the proteolytic enzyme with up to two allowed missed cleavage sites. Quantification based on TMT-10plex MS3 reporter ion intensity was enabled. The results were filtered for a minimal length of seven amino acids and a 1% PSM and protein FDR. MaxQuant results were imported into the software suite Perseus[5] (v.1.5.8.5). TMT intensities per sample were normalized for median of total TMT reporter intensities across all samples and imported into Perseus v.1.5.8.5, followed by categorical annotation of the treatments „Ctrl", „PDPm-*Nal*", and „PDP-*Nal*". Next, the dataset of 8,477 phosphorylation-sites was reduced to 7,199 by only retaining class I phosphorylation-sites, i.e. sites with a localization probability >0.75. Based on this dataset, pairwise comparisons were carried out by a two-sided unpaired Student's t-test adjusted for multiple-hypothesis testing (250 randomizations, FDR 5%, S0: 0.1). Results were exported and final plots of only pSer/pThr sites were produced in R using ggplot2 and the tidyverse package. Sites and proteins were compared against

PPP1CA, PPP1CB and PPP1CC substrates reported in DEPOD[9] (accessed May 2023) as well as to those annotated as PPP1R in the HGNC database[8] and those reported to bind PP1 through an RVxF motif in the KVxF PP1 docking motif repository.[10]

1.3.4. Recombinant PP1α treatment on immunoprecipitated IRS2-mVenus and sample preparation for phosphoproteomics

Culturing, immune-precipitation and in vitro treatment of IRS2-mVenus with recombinant PP1α. Recombinant PP1α was expressed and purified as reported by Hoermann et al.[1] HeLa Kyoto cells (BIOSS cell line repository) were grown to 70% confluency in five 6 cm cell culture dishes in general growth medium (DMEM, 4.5 g/L glucose, GlutaMAX™, 25 mM HEPES, Gibco) and transfected with pcDNA-FRT/TO-IRS2-mVenus. Transfection was carried out following the manufacturer's instructions using 3 µg plasmid DNA in 250 µL Opti-MEM (Gibco) and 15 µL FuGene HD (Promega) per plate (1 plate per replicate). 24 h after transfection, cells were incubated with 20 nM Calyculin A (# 9902 S, Cell Signaling Technology) in serum-free medium (DMEM, low glucose, GlutaMAX™, Gibco) for 12 min at 37 °C. Cells were placed on ice and washed twice with 5 mL cold PBS before 300 µL lysis buffer (100 mM NaCl, 10 mM Tris-HCl pH 7.5, 0.1% IGEPAL, 1 mM EDTA, 1 mM EGTA, 20 nM Calyculin A, 1x cOmplete Mini protease inhibitor cocktail (#04693132001, Roche), 1x PhosSTOP phosphatase inhibitor cocktail (Sigma), 2 mM DTT, 2 mM $MnCl_2$) was added. Cells were scraped from the plates, lysed by 10 pushes through an injection needle and the insoluble fraction was pelleted by centrifugation (10 min, 10^4 rcf, 4 °C). From the resulting supernatant, 20 µL were removed as input sample. The remainder was used for immunoprecipitation. The lysate from each plate was incubated with 15 µL magnetic agarose GFP-Trap beads (Chromotek, prewashed in lysis buffer) in 1.5 mL Eppendorf tubes on a rotation wheel at 4 °C for 2 h. After 2 h incubation, each tube was exposed to magnetic field and the supernatant was removed. After two washes with 500 µL lysis buffer, the magnetic beads from each tube with bound IRS2 were resuspended in 500 µL lysis buffer and split into two tubes, one for treatment with PP1 and one left untreated as a control. The lysis buffer was discarded and beads were incubated with 300 nM recombinant mVenus-PP1α in 30 µL lysis buffer or only buffer for the controls for 30 min at 30 °C. Tubes were removed from the heat block and gently agitated every 10 minutes to resuspend the beads. Samples were eluted by the addition of 30 µL 2x reducing SDS sample buffer (125 mM Tris-HCl pH 6.8, 20% v/v glycerol, 4% w/v SDS, 0.02% w/v bromophenol blue, 5% v/v β-mercaptoethanol, 1 mM DTT) to the buffer and incubation of the bead slurry at 95 °C for 10 min. Samples were separated using 10-well NuPAGE™ 4 to 12% Bis-Tris 1.0 mm mini protein gels (Invitrogen) in MOPS buffer at 135 V for 1.5 h and analyzed by Coomassie Staining. The bands at 212 kDa, corresponding to the IRS2-mVenus fusion protein, were cut from the gel and subsequently sent to MS analysis.

In-gel trypsin digestion of all 212 kDa gel bands was performed according to standard procedures.[2] Briefly, protein bands were reduced (50 mM dithiothreitol), alkylated (55 mM chloroacetamide) and digested overnight with trypsin (Trypsin Gold, mass spectrometry grade, Promega). The peptides obtained were dried to completeness and resuspended in 12 µL of buffer A (2% acetonitrile, 0.1% formic acid in HPLC grade water). 5 µL of sample were injected per MS measurement.

Mass spectrometric data acquisition for phosphoproteomics of IRS2-mVenus dephosphorylation.

LC-MS/MS data acquisition was carried out on a Dionex Ultimate 3000 RSLCnano system coupled to a Q-Exactive HF-X mass spectrometer (Thermofisher Scientific, Bremen). Injected peptides were delivered to a trap column (ReproSil-pur C18-AQ, 5 µm, Dr. Maisch, 20 mm × 75 µm, self-packed) at a flow rate of 5 µL/min in 100% solvent A (0.1% formic acid in HPLC grade water). After 10 minutes of loading, peptides were transferred to an analytical column (ReproSil Gold C18-AQ, 3 µm, Dr. Maisch,

450 mm × 75 µm, self-packed) and separated using a 50 min gradient from 4% to 32% of solvent B (0.1% FA, 5% DMSO in acetonitrile) in solvent A (0.1% FA, 5% DMSO in HPLC grade water) at 300 nL/min flow rate. The Q-Exactive HF-X mass spectrometer was operated in data dependent acquisition (DDA) and positive ionization mode. MS1 spectra (360–1300 m/z) were recorded at a resolution of 60k using an automatic gain control (AGC) target value of 3e6 and maximum injection time (maxIT) of 45 ms. Up to 18 peptide precursors were selected for fragmentation. Only precursors with charge state 2 to 6 were selected and dynamic exclusion of 25 s was enabled. Peptide fragmentation was performed using higher energy collision induced dissociation (HCD) and a normalized collision energy (NCE) of 26%. The precursor isolation window width was set to 1.3 m/z. MS2 Resolution was 15.000 with an automatic gain control (AGC) target value of 1e5 and maximum injection time (maxIT) of 25 ms.

Data analysis for phosphoproteomics of IRS2-mVenus dephosphorylation.

In a first step, the recoded LC-MS/MS RAW files were analyzed using the MaxQuant software[3] (version 1.6.3.4) with its built-in search engine Andromeda[4]. MS2 spectra were searched against the canonical Uniprot human reference database (UP000005640, download from Uniprot July 2020, 20353 protein entries) supplemented with common contaminants. Oxidation of methionine, acetylation at the protein N-terminus and phosphorylation on Ser, Thr, and Tyr were specified as variable modifications. Carbamidomethylation on cysteines was specified as a fixed modification. Trypsin (Trypsin/P) specified as the proteolytic enzyme with up to two allowed missed cleavage sites. Results were filtered for a minimal length of seven amino acids and a 1% PSM and protein FDR. The quantitative phosphosite-specific results (Phospho (STY)Sites.txt) were imported into the software suite Perseus[5] (v.1.5.8.5). The dataset of 192 phosphorylation-sites was reduced to 82 sites after removing reverse and contaminant peptides and after filtering for "valid values" in 3 out of 5 replicates in at least one group. Out of the 82 detected phospho-sites 73 were IRS2-derived sites. (Phospho)peptides containing phosphosites that showed differences between with and without imputation in Perseus4 (v.1.5.8.5), as well as some phosphopeptides for which we could not observe any significantly regulation, were obtained as a list for further analysis.

In a second step, the 73 IRS2-derived (phospho)peptides were manually validated using the targeted data analysis software Skyline[16] (64-bit, v.20.2.0.343). This included manual spectrum and chromatogram inspection as well as manually peak integration. For that, the 73 targeted (phospho)peptides of IRS2 were defined within Skyline, followed by the DDA RAW file import. MS2 identifications were visualized for each raw file specifically by generating a redundant spectral library using the MaxQuant search results described above. The corresponding Skyline document can be accessed and visualized via the Panorama Public repository[17] as described in the data availability statement. Based on the (phospho)peptide-specific MS1 chromatogram intensities exported from Skyline, mass spectrometric intensity values were obtained. Comparison of untreated and PP1α treated groups was determined by using a two-sided unpaired multiple t-test (p-value < 0.05) in GraphPad Prism 6. Significant dephosphorylation was detected for singly phosphorylated peptides including pSer342 (p-value: 0,0011193 , fold-change: 4.9), pSer346 (p-value: 1,130975e-005, fold-change: 9.6) and pSer391 (p-value: 0,00774617 , fold-change: 3.5), as well as for the doubly dephosphorylated peptides including ppSer346-Thr350 (p-value: 0,000134109, fold-change: 6.8) and ppSer384-Ser391 (p-value: 0,00116275, fold-change: 4.9).

1.4. Data analysis and integration of in lysate dephosphorylation experiment reported previously

Data analysis for Phosphoproteomics of dephosphorylation of HeLa cell lysates by recombinant PP1α. Data obtained from the experiment reported by Hoermann *et al.*[1] was analyzed for total intensities of p-sites. Following the analysis described in section "Phosphoproteomic data analysis" of the article by Hoermann *et al.*[1], the median-normalized data was filtered for reverse peptides and potential contaminants using the software suite Perseus[5] (version 1.5.8.5). The dataset was then processed further using R. The normalized intensities of singly, doubly and triply phosphorylated peptides for each site after PP1 treatment or untreated control were summed to give the total intensities. Next, the dataset of 3,230 phosphorylation-sites was reduced to 3,090 by only retaining class I phosphorylation-sites, i.e. sites with a localization probability >0.75. This filtered dataset was again imported into Perseus, followed by categorical annotation of treatment conditions PP1 or Ctrl. Based on this dataset, conditions were compared by a two-sided unpaired Student's t-test (250 randomizations, FDR 5%, S0: 0.1). Results were exported and final plots of only pSer/pThr sites were produced in R using ggplot2 and the tidyverse package.

Integration of three phosphoproteomics datasets. Data obtained from t-tests from the three Phosphoproteomics experiments described above (PDP treatment, in vitro dephosphorylation of lysates, in vitro dephosphorylation of IRS2-mVenus) were combined using R and the tidyverse package. The dataset obtained from PDP treatment was used and information on whether p-sites were significantly decreased after the two in vitro dephosphorylation experiments was added. Sites were compared using the sequence window ±15 amino acids around the p-sites.

2. Supplementary figures

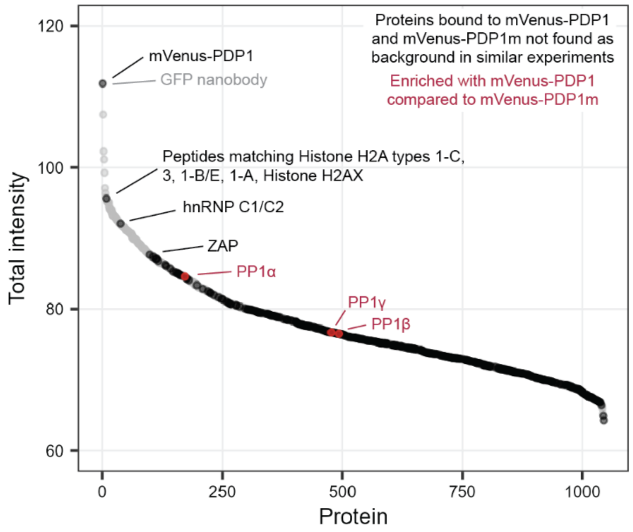

Figure S1. *Supplementary figure for immunoprecipitation experiment with mVenus-PDP1(m) shown in Figure 1A. Sum of intensities of proteins bound to mVenus-PDP1m. Proteins significantly different between mVenus-PDP1 and mVenus-PDP1m highlighted in red, proteins that are not found as background in similar experiments in black, with proteins among the 100 highest intensities labeled.*

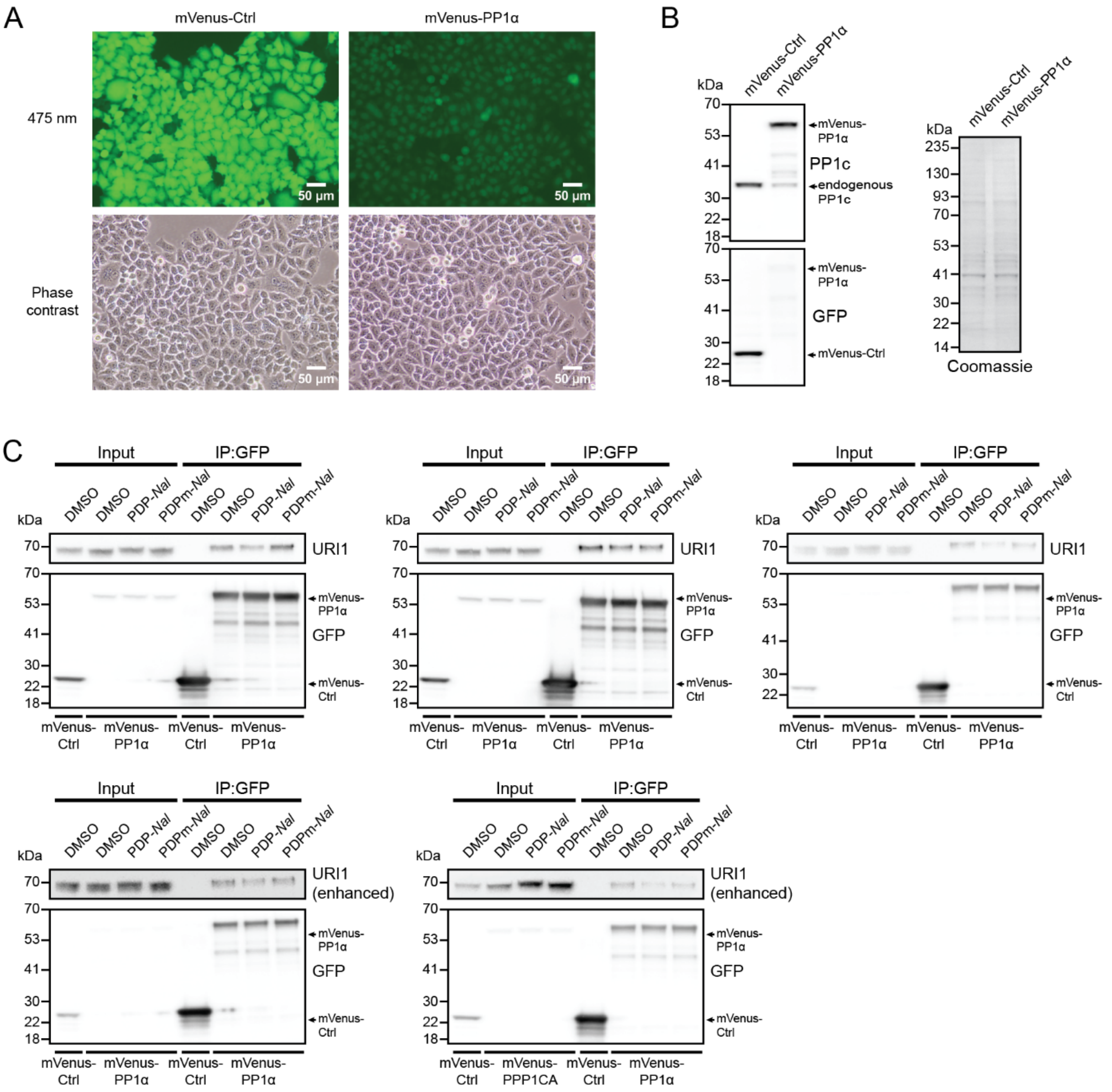

Figure S2. *Supplementary figure for immunoprecipitation experiment with mVenus-Ctrl and mVenus-PPP1CA using stable cell lines. A) Microscopy images (fluorescence after irradiation at 475 nm and phase contrast) of HeLa FlpIn cells expressing mVenus-Ctrl or mVenus-PPP1CA after 24 h induction with 1 µg/mL doxycycline. Images were recorded with identical settings for both cell lines using a 10X objective. Scale bar 50 µm. B) Immunoblots and colloidal Coomassie stained SDS-gel of lysates of HeLa FlpIn cells expressing mVenus-Ctrl or mVenus-PPP1CA after 24 h induction with 1 µg/mL doxycycline. C) Five replicates of the immunoprecipitation experiment shown in Figure 2A to assess changes in binding of URI1 to mVenus-PPP1CA. Unmodified blots were used for quantification together with the replicate shown in Figure 2E.*

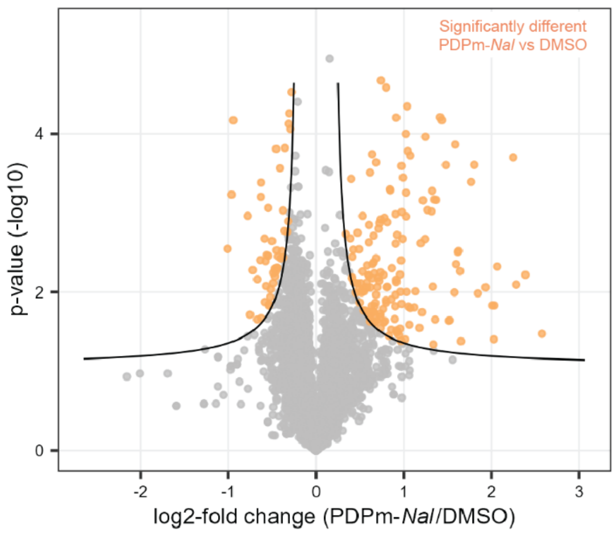

Figure S3. *Supplementary figure for phosphoproteomics experiment after PDP(m)-Nal treatment shown in Figure 3B, comparing pSer/pThr phosphosites after PDPm-Nal treatment and DMSO treatment. 204 phosphosites significantly different after PDPm-Nal treatment compared to DMSO treatment highlighted in orange. Significance threshold: FDR = 0.05, s0 = 0.1.*

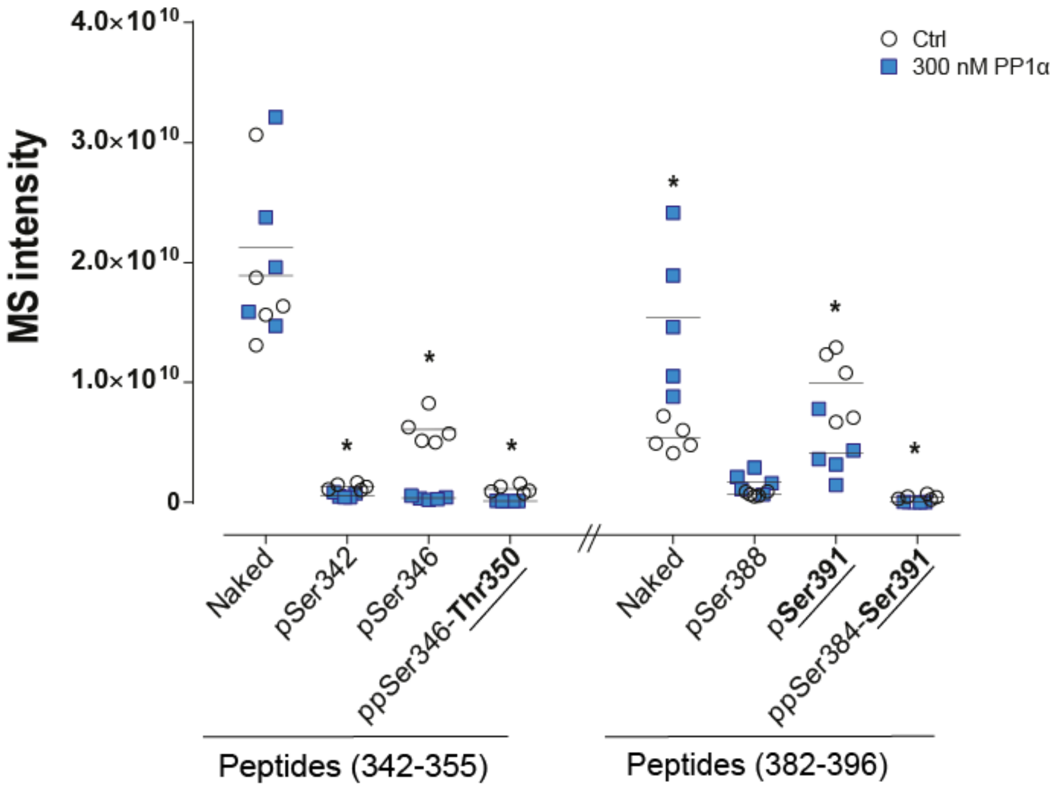

Figure S4. *Supplementary figure for phosphoproteomic experiment in the in vitro dephosphorylation assay of IRS2-mVenus shown in Figure 4E. This graph shows the full y-axis version of Figure 4E, where it is shown in two segments to better illustrate the differences between Ctrl and 300 nM PP1α for phosphopeptides with low MS intensities. * p-value < 0.05; n=5.*

3. Raw images of Western blots

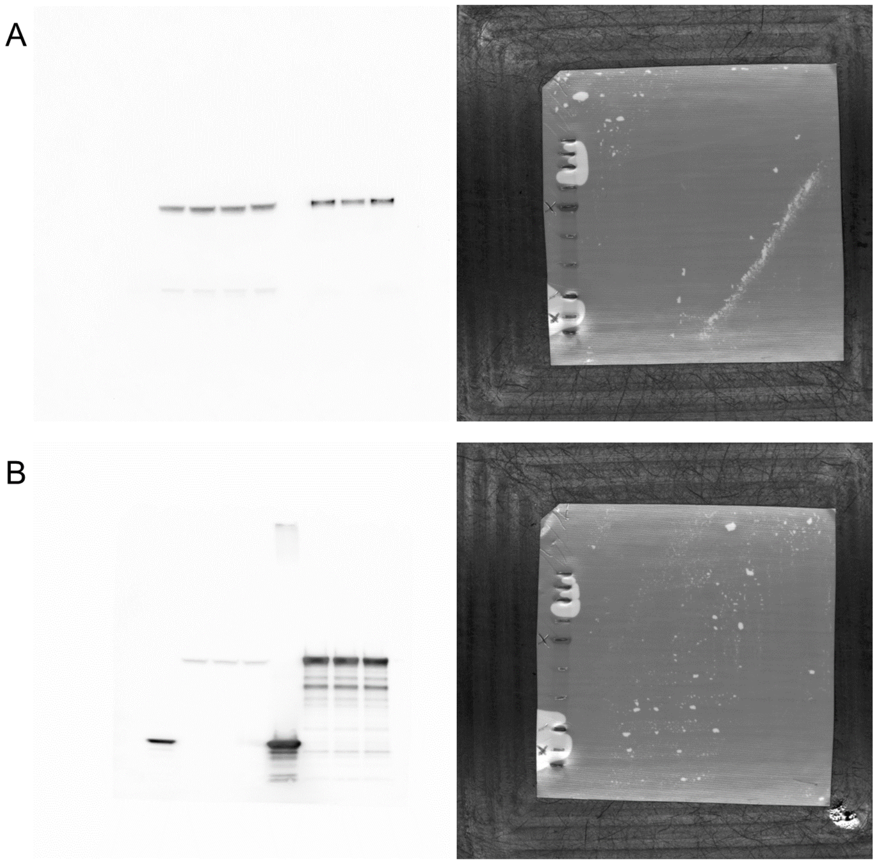

Raw images of blots shown in Figure 2E. A) α-URI blot and marker, B) α-GFP blot and marker.

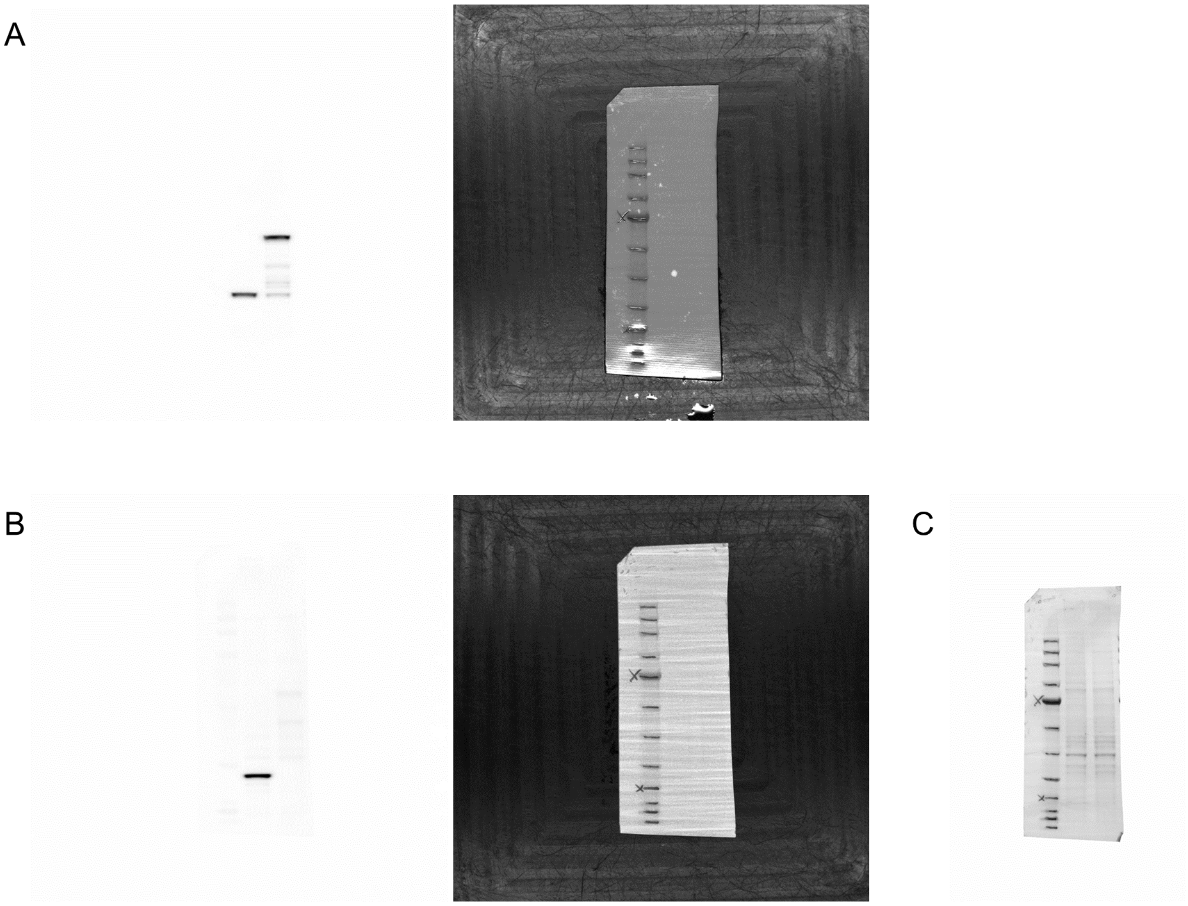

Raw images of blots shown in Figure S2B. A) α-PP1c blot and marker, B) α-GFP blot and marker, C) Coomassie-stained membrane.

A

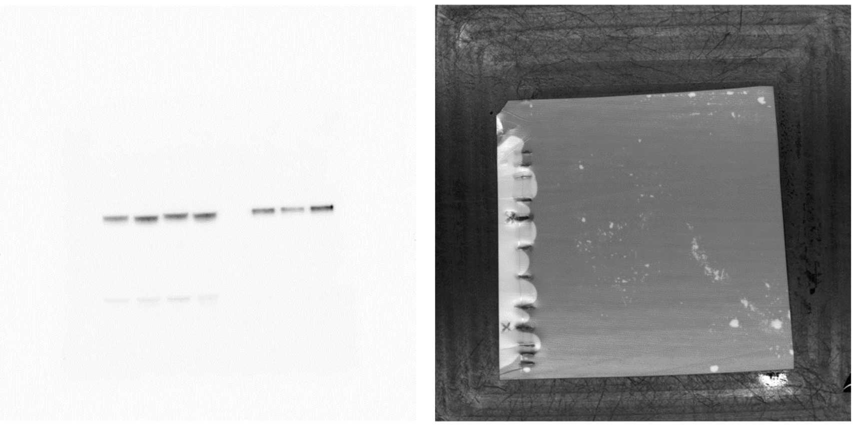

B

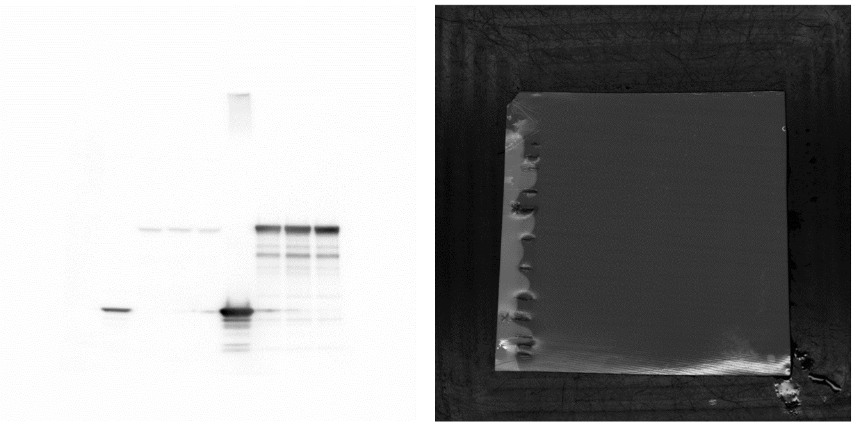

C

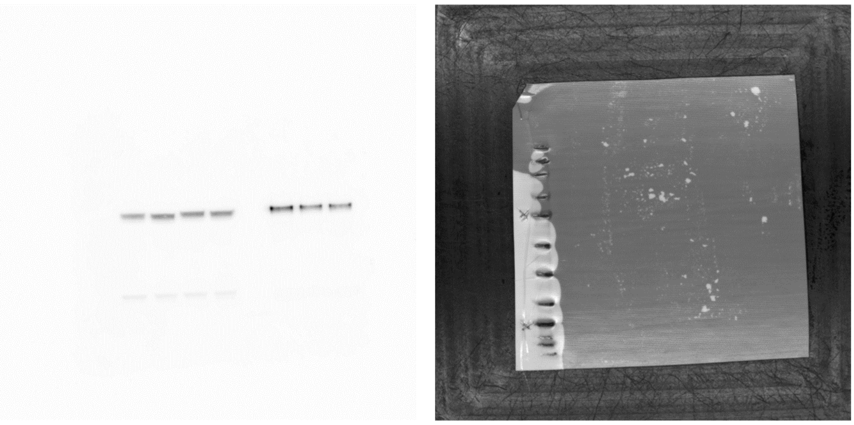

D

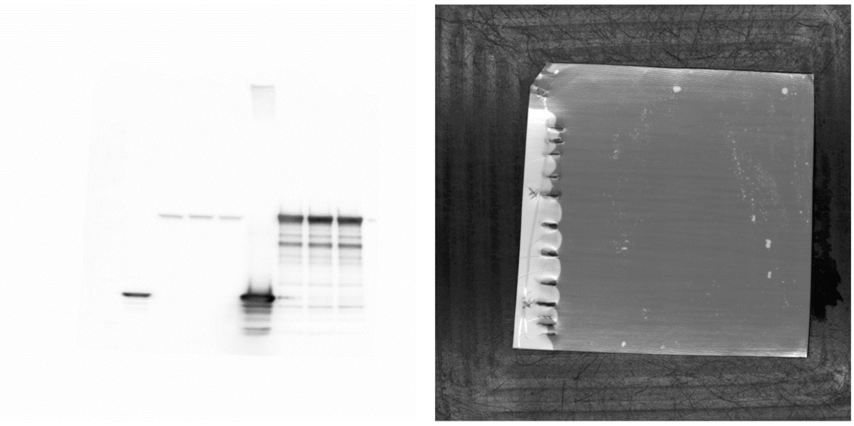

E

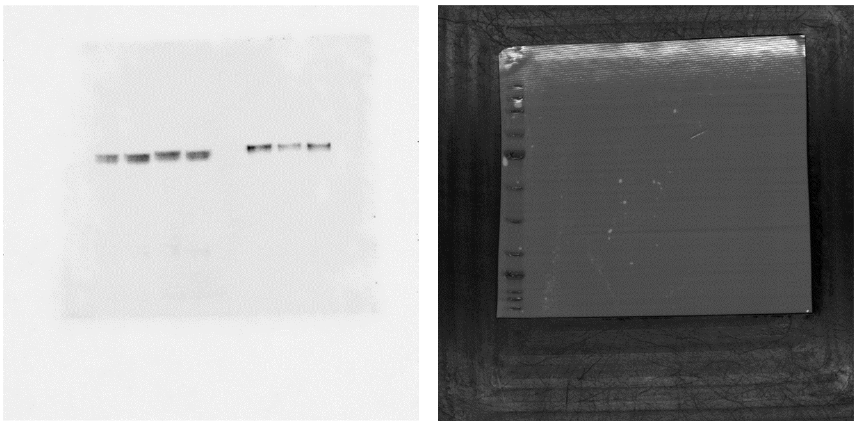

F

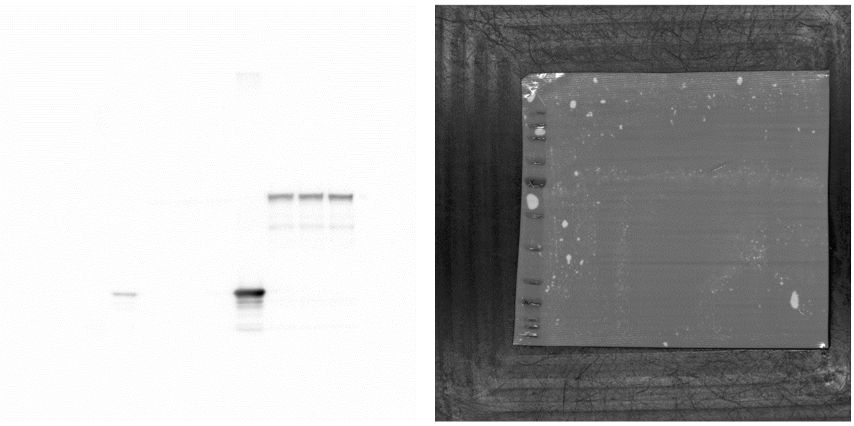

G

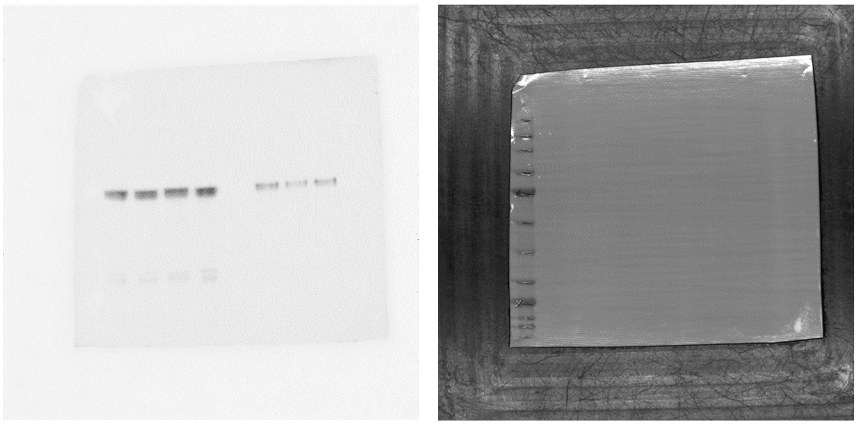

H

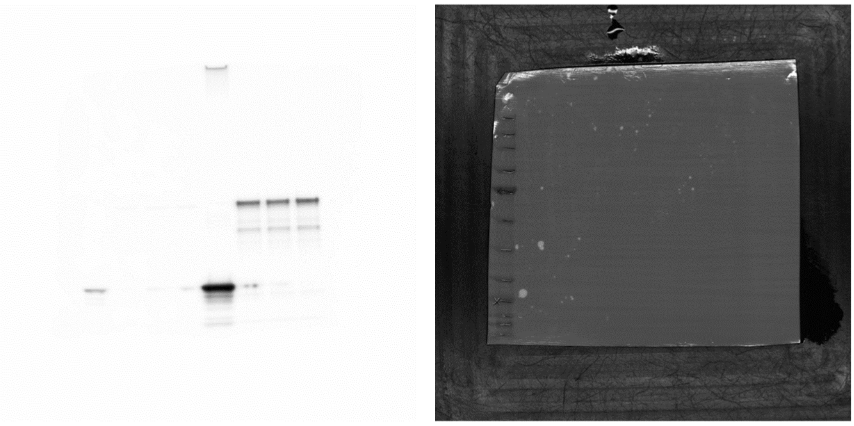

I

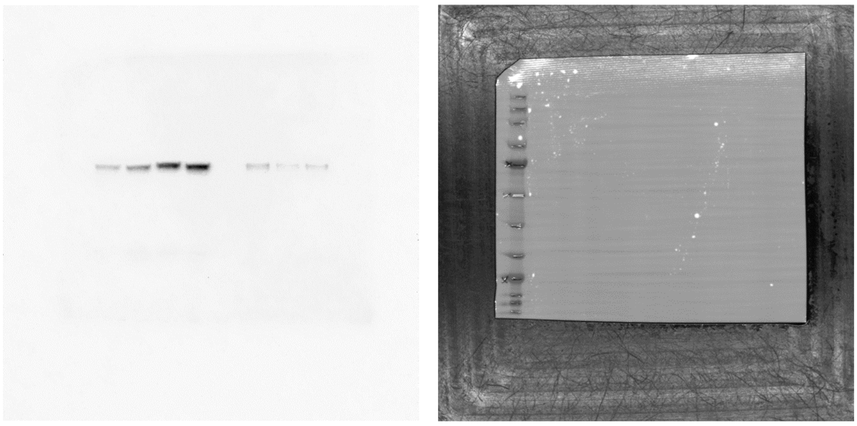

J

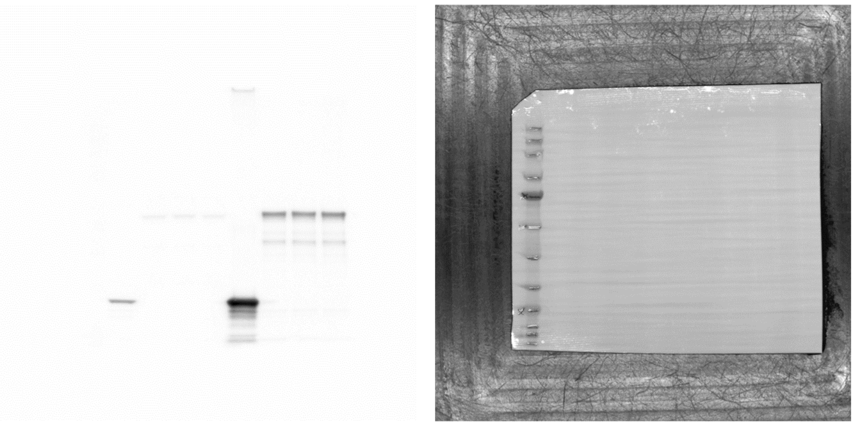

Raw images of blots shown in Figure S2C. A), C), E), G) and I) α-URI blots and markers, B), D), F), H) and J) α-GFP blots and markers.

4. Description of additional supplementary materials

Supplementary table 1: Supp table 1 IP mVenus-PDP1(m).xlsx

MS data from immunoprecipitation of mVenus-PDP1 and mVenus-PDP1m, processed and filtered as described above in the section on data analysis.

Sheet 1: Total intensities for each protein were compared between mVenus-PDP1 and mVenus-PDP1m using a t-test.

Sheet 2: Summed intensities of all three replicates (for proteins which were found in all three replicates). Annotated for whether proteins were also enriched with mVenus-Ctrl (Supp. Table 2) or other affinity purification MS (AP-MS) experiments using HeLa cells listed in the CRAPome database[6] to identify proteins that bind to PDPs outside the RVTF motif and determine their abundance.

Supplementary table 2: Supp table 2 IP mVenus-PP1alpha.xlsx

MS data from immunoprecipitation of mVenus-PP1α treated with PDP-*Nal* or PDPm-*Nal* and mVenus-Ctrl, processed and filtered as described above in the section on data analysis. Total intensities were compared between mVenus-PP1α after PDPm-*Nal* treatment and mVenus-Ctrl ("PDPm-*Nal* vs Ctrl") and mVenus-PP1α after PDP-*Nal* and PDPm-*Nal* treatment ("PDP-*Nal* vs PDPm-*Nal*") using t-tests. Annotated for whether proteins are known interactors of PP1c or part of the PAQosome.

Supplementary table 3: Supp table 3 phosphoproteomics.xlsx

MS data from three phosphoproteomics experiments, processed and filtered as described above in the section on data analysis.

Sheet 1: PDP treatment of HeLa cells: Total intensities for each p-site after PDP-*Nal*, PDPm-*Nal* or DMSO treatment were processed as above and compared using t-tests. Annotated for whether sites are known PP1 substrate sites, are located on a known PP1 substrate with unknown site, are located on a known interactor of PP1c, are dephosphorylated by PP1 in vitro in lysate or after IP in the case of IRS2.

Sheet 2: In vitro dephosphorylation of HeLa lysates using recombinant PP1α: Total intensities for each p-site of previously published data were processed as described above and compared between PP1 treatment and untreated lysates using a t-test.

Sheet 3: High confidence substrate candidates arising from the comparison of the two Phosphoproteomics experiments. 315 p-sites that change significantly following PDP-*Nal* treatment and are also dephosphorylated by PP1α in vitro.

Sheet 4: Skyline label free intensities of IRS2 phosphopeptides for amino acids 342-355 and 382-396 used for a two-sided unpaired multiple t-test.

5. References

1 B. Hoermann, T. Kokot, D. Helm, S. Heinzlmeir, J. E. Chojnacki, T. Schubert, C. Ludwig, A. Berteotti, N. Kurzawa, B. Kuster, M. M. Savitski and M. Köhn, Dissecting the sequence determinants for dephosphorylation by the catalytic subunits of phosphatases PP1 and PP2A, *Nat. Commun.*, 2020, **11**, 3583.

2 A. Shevchenko, H. Tomas, J. Havli, J. V. Olsen and M. Mann, In-gel digestion for mass spectrometric characterization of proteins and proteomes, *Nat Protoc*, 2006, **1**, 2856–2860.

3 S. Tyanova, T. Temu and J. Cox, The MaxQuant computational platform for mass spectrometry-based shotgun proteomics, *Nat. Protoc.*, 2016, **11**, 2301–2319.

4 J. Cox, N. Neuhauser, A. Michalski, R. A. Scheltema, J. V. Olsen and M. Mann, Andromeda: A Peptide Search Engine Integrated into the MaxQuant Environment, *J. Proteome Res.*, 2011, **10**, 1794–1805.

5 S. Tyanova, T. Temu, P. Sinitcyn, A. Carlson, M. Y. Hein, T. Geiger, M. Mann and J. Cox, The Perseus computational platform for comprehensive analysis of (prote)omics data, *Nat. Methods*, 2016, **13**, 731–740.

6 D. Mellacheruvu, Z. Wright, A. L. Couzens, J.-P. Lambert, N. St-Denis, T. Li, Y. V. Miteva, S. Hauri, M. E. Sardiu, T. Y. Low, V. A. Halim, R. D. Bagshaw, N. C. Hubner, A. al-Hakim, A. Bouchard, D. Faubert, D. Fermin, W. H. Dunham, M. Goudreault, Z.-Y. Lin, B. G. Badillo, T. Pawson, D. Durocher, B. Coulombe, R. Aebersold, G. Superti-Furga, J. Colinge, A. J. R. Heck, H. Choi, M. Gstaiger, S. Mohammed, I. M. Cristea, K. L. Bennett, M. P. Washburn, B. Raught, R. M. Ewing, A.-C. Gingras and A. I. Nesvizhskii, The CRAPome: a Contaminant Repository for Affinity Purification Mass Spectrometry Data, *Nat. Methods*, 2013, **10**, 730–736.

7 G. Candiano, M. Bruschi, L. Musante, L. Santucci, G. M. Ghiggeri, B. Carnemolla, P. Orecchia, L. Zardi and P. G. Righetti, Blue silver: A very sensitive colloidal Coomassie G-250 staining for proteome analysis, *Electrophoresis*, 2004, **25**, 1327–1333.

8 R. L. Seal, B. Braschi, K. Gray, T. E. M. Jones, S. Tweedie, L. Haim-Vilmovsky and E. A. Bruford, Genenames.org: the HGNC resources in 2023, *Nucleic Acids Res.*, 2023, **51**, D1003–D1009.
9 N. P. Damle and M. Köhn, The human DEPhOsphorylation Database DEPOD: 2019 update, *Database*, 2019, **2019**, baz133.
10 KVxF PP1 motif repository, http://slim.icr.ac.uk/motifs/pp1/index.php?page=instances, (accessed 26 May 2023).
11 Western blot membrane stripping for restaining protocol | Abcam, https://www.abcam.com/protocols/western-blot-membrane-stripping-for-restaining-protocol, (accessed 26 May 2023).
12 J. Zecha, S. Satpathy, T. Kanashova, S. C. Avanessian, M. H. Kane, K. R. Clauser, P. Mertins, S. A. Carr and B. Kuster, TMT Labeling for the Masses: A Robust and Cost-efficient, In-solution Labeling Approach, *Mol. Cell. Proteomics*, 2019, **18**, 1468–1478.
13 B. Ruprecht, H. Koch, G. Medard, M. Mundt, B. Kuster and S. Lemeer, Comprehensive and Reproducible Phosphopeptide Enrichment Using Iron Immobilized Metal Ion Affinity Chromatography (Fe-IMAC) Columns, *Mol. Cell. Proteomics*, 2015, **14**, 205–215.
14 B. Ruprecht, J. Zecha, D. P. Zolg and B. Kuster, in *Proteomics: Methods and Protocols*, eds. L. Comai, J. E. Katz and P. Mallick, Springer, New York, NY, 2017, pp. 83–98.
15 G. C. McAlister, D. P. Nusinow, M. P. Jedrychowski, M. Wühr, E. L. Huttlin, B. K. Erickson, R. Rad, W. Haas and S. P. Gygi, MultiNotch MS3 Enables Accurate, Sensitive, and Multiplexed Detection of Differential Expression across Cancer Cell Line Proteomes, *Anal. Chem. (Washington, DC, U. S.)*, 2014, **86**, 7150–7158.
16 B. MacLean, D. M. Tomazela, N. Shulman, M. Chambers, G. L. Finney, B. Frewen, R. Kern, D. L. Tabb, D. C. Liebler and M. J. MacCoss, Skyline: an open source document editor for creating and analyzing targeted proteomics experiments, *Bioinformatics*, 2010, **26**, 966–968.
17 V. Sharma, J. Eckels, B. Schilling, C. Ludwig, J. D. Jaffe, M. J. MacCoss and B. MacLean, Panorama Public: A Public Repository for Quantitative Data Sets Processed in Skyline *, *Molecular & Cellular Proteomics*, 2018, **17**, 1239–1244.
